# Capturing inter-individual variability in stress dynamics with heart rate traces reveals activity in the bilateral hippocampus, amygdala, and insula

**DOI:** 10.1162/imag_a_00376

**Published:** 2024-12-02

**Authors:** Mira Erhart, Anne Kühnel, Tanja M. Brückl, Julia Fietz, Dorothee Pöhlchen, Natan Yusupov, Immanuel Elbau, Michael Czisch, Elisabeth B. Binder, Philipp G. Sämann, Victor I. Spoormaker

**Affiliations:** Department of Genes and Environment, Max-Planck-Institute of Psychiatry, Munich, Germany; International Max Planck Research School for Translational Psychiatry, Munich, Germany; Section of Medical Psychology, Department of Psychiatry and Psychotherapy, Faculty of Medicine, University of Bonn, Bonn, Germany; Weill Cornell Medical College, New York City, NY, United States; Max-Planck-Institute of Psychiatry, Munich, Germany

**Keywords:** fMRI, stress, amygdala, hippocampus, insula, heart rate

## Abstract

Abnormalities in the neural mechanisms of the stress response have the potential to serve as a transdiagnostic marker for stress-related disorders. This potential is rooted in the highly individual and dynamic stress response, which poses a challenge to classical experimentally informed models that focus on specific phases, conditions, or stimuli during a stress task. Here, we integrate individual heart rates (HR) as an immanent index of the stress-response and combine these with functional magnetic resonance imaging (fMRI) data. In this study, 83 healthy participants completed a multimodal psychosocial imaging stress task comprising three different task phases (*PreStress*,*Stress,*and*PostStress*), with each phase consisting of five 60 s blocks of active task interleaved with 40 s of rest, and simultaneous recordings of pulse plethysmography. Participants were asked to solve mental calculations and were exposed to negative social feedback during the*Stress*phase. We estimated a general linear model (GLM) with individual heart rates averaged per active block as a single parametric modulator of the task regressor across all 15 active blocks irrespective of the task phase. Results revealed a negative correlation between HR and activation in the bilateral amygdala and anterior hippocampus as well as deactivation in the default mode network. Positive correlations with HR were detected in the bilateral insular cortex, bilateral angular gyrus, and parts of the inferior and superior parietal lobes. In summary, our findings emphasize the utility of integrating the commonly assessed cardiovascular stress response (here: HR) as an immediate index of the participant’s stress status. We conclude that by such integration, brain regions involved in regulating the acute stress response, such as the anterior hippocampus and amygdala, are detected more sensitively by tracking the individual’s “stress wave” rather than treating every experimental block uniformly. Our approach may serve as a complementary analysis to the task-regressor based model.

## 1 Introduction

Abnormal responses to psychological stress are a hallmark of stress-related psychiatric disorders (Aldao et al., 2010;van Oort et al., 2020;White et al., 2014;Zorn et al., 2017). The concept of stress, which was introduced by Hans Selye (Selye, 1998), has been described as an unspecific bodily adaption to an external homeostatic challenge (Bali & Jaggi, 2015;Rochette et al., 2023) and has frequently been studied in humans with psychological stressors that lead to negative affect and constitutes high cognitive task demands (Bali & Jaggi, 2015;Noack et al., 2019).

The adaptions to a stressor are initiated on the neural level and contain two systems: first, the rather immediate-responding autonomous nervous system (ANS) and second, the cortical stress systems, which mainly address the HPA axis, resulting in the secretion of cortisol in humans (Joels et al., 2013;Ziegler, 2012).

Firstly, the ANS responds immediately to stressors through the sympathetic nervous system (SNS), triggering blood pressure and increase in HR without cortical processing (Schwabe et al., 2022). A major source of primary efferents that regulates the ANS originate from hypothalamic nuclei. These nuclei receive input from various limbic and cortical regions, including the centromedial nucleus of the amygdala and the insula (Johnson, 2013;Lamotte et al., 2021;Wehrwein et al., 2016). Its initial response to a stressor is fast, but not long-lasting (Schwabe et al., 2022). If a stressor persists, the HPA-axis is activated before the cessation of the ANS response (Schwabe et al., 2022). Its activation results in numerous consequences aimed at optimizing the body’s adaptation to a stressor (Joels et al., 2013), including a negative feedback loop to downregulate the stress response after successful adaption (Herman et al., 2003,^2016^). This homeostatic balance is established by the interplay of many different brain regions, including the hippocampus, amygdala, prefrontal cortex (PFC), hypothalamus, and lateral septum (Herman et al., 2003,^2020^).

Various neuroimaging techniques have been used to study the neural correlates of the human stress response such as functional magnetic resonance imaging (fMRI) studies in a variety of different stress tasks (Berretz et al., 2021). One commonly used task is the Montreal Imaging Stress Task (MIST), which combines mental calculations challenges with social evaluative feedback as generally effective and well-standardizable stressors (Dedovic et al., 2005). Over the years, the MIST has been studied in both healthy controls and patient cohorts with different conditions such as obsessive-compulsive disorder, bipolar disease, or major depressive disorder (Lord et al., 2012;Villarreal et al., 2021). Other studies focused on the lasting impact of stressful life events such as childhood maltreatment, or trait anxiety as a known source of psychiatric risk (Chung et al., 2016;Corr et al., 2021;Orem et al., 2019;Wheelock et al., 2016;Zhong et al., 2019) and employed different analytical approaches, for example, blockwise and event-related regressors (Chung et al., 2016;Dedovic, Rexroth, et al., 2009). Common responses to stress frequently included activations observed in the insula, the anterior and posterior cingulate cortex, parts of the PFC, such as the dorsolateral PFC (DLPFC), as well as deactivations of the hippocampus (Dedovic, D’Aguiar, & Pruessner, 2009;Noack et al., 2019).

**Fig. 1.**
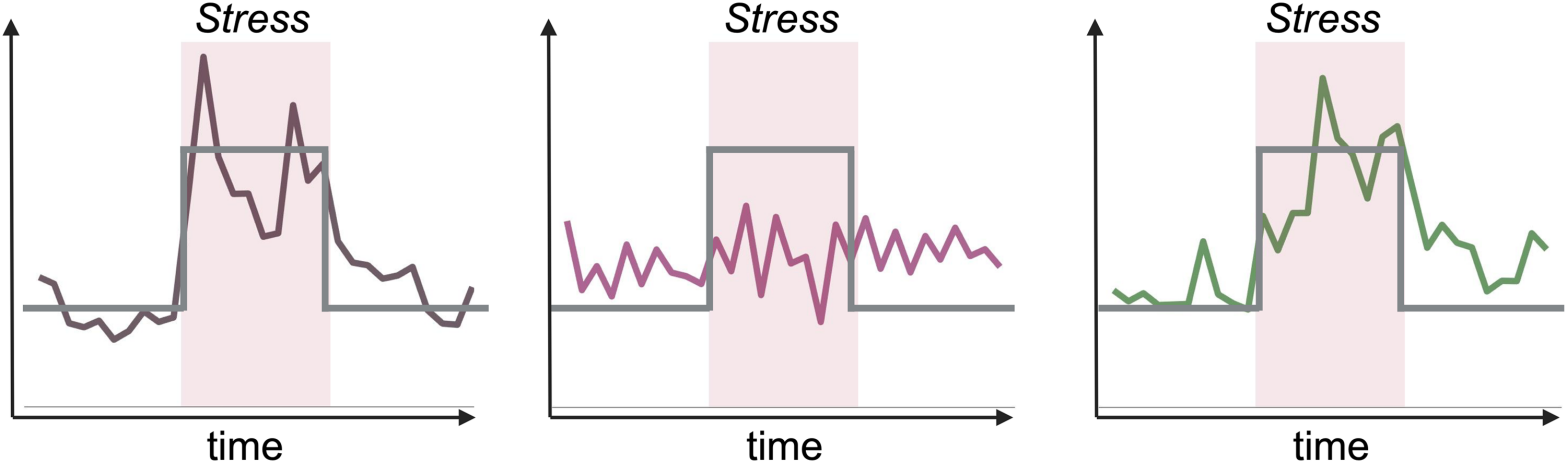
Illustration of the study purpose: refined capturing of the individual stress wave by incorporation of heart rate traces. Three example heart rates traces (brown, pink, green) throughout the whole stress experiment are illustrated together with the classical box-car regressors (grey) contrasting each StressPhase uniformely against the Pre and PostStress phases without individual weighting. The differences among the individual’s rate traces illustrate the high interindividual variability and stress dynamic. This figure has been modified withBioRender.com.

In a meta-analysis spanning various fMRI stress-tasks,Berretz et al. (2021)found significant stress-related activation in the bilateral insula, the claustrum, and the inferior frontal gyrus, and deactivation in the right amygdala and the parahippocampal gyrus. Aside from the activation in the inferior frontal gyrus, which putatively reflects general task-related processes such as mental calculations, the other regions were interpreted as being specifically involved in the stress-response or regulators of the stressors’ emotional consequences. Convergent with these findings, the bilateral anterior insula has been implicated in both responses to psychosocial and physiological stressors as reported by meta-analytic stress studies (Kogler et al., 2015;Qiu et al., 2022). Even though these meta-analytic studies provide a useful summary of stress-related brain responses across different stress tasks and appear to converge towards insular activity associated with stressors, the pattern of activation or deactivation of the amygdala and the hippocampus is more ambiguous. A plausible reason for the somewhat low consistency in the limbic regions may lie in the fast-changing dynamics of the stress response in general (Elbau et al., 2018) and individual differences in stress-related brain activity (Chung et al., 2016;Henckens et al., 2016). As described before, most studies used repetitive mental calculation blocks and negative social feedback as stressors (Dedovic, D’Aguiar, & Pruessner, 2009;Dedovic, Rexroth, et al., 2009). The fluctuations in stress reactivity may be more variable than just being temporally aligned with longer experimental blocks or phases (Chung et al., 2016;Zhong et al., 2019). Technically, in the reported imaging stress studies, the regressors representing the high pressure/stress blocks treat these blocks uniformly without flexible weighting (Fig. 1). This may desensitize the analysis due to factors of individuality and the fast-changing dynamics of the stress response.

Such temporal fluctuations of the fMRI correlate of the stress response can, indeed, be detected with temporally fine-grained models applied to functional connectivity (Kühnel et al., 2022) or hemodynamic response functional markers (Elbau et al, 2018). Another option is to directly embed peripheral stress indices into the statistical fMRI models of the imaging stress task, such as the widely available HR measured with pulse photoplethysmography (PPG) or electrocardiography (ECG). Measures of HR variability (HRV) also have been shown to identify periods of mental stress (Vaishali et al., 2022) with a high inter-individual variability (Singh et al., 2018), which may potentially capture individual stress peaks (Fig. 1).

In 2009,Gray et al. (2009)reviewed the use of several peripheral physiological measures in fMRI research over the preceding years. One implementation of such a peripheral marker into the general linear model has been done byOrem et al. (2019), who recorded skin conductance (SCR) and HR during the MIST and used differential values (control – stress) in the second-level analysis. Activity in various prefrontal cortex (PFC) regions and the amygdala varied depending on the SCR response.Wager et al. (2009)used another stress task in which participants had to prepare a speech on a given topic and calculated the mediating effect of brain activity for experimental manipulations on cardiovascular responsiveness using a multi-level path model. Results of the study identified two distinct medial PFC regions, whose coupling with the experimental stressor predicted cardiovascular stress responsiveness.Gianaros et al. (2005)andCausse et al. (2022)used the arterial blood pressure/HR of each participant as a parametric modulation in the first-level models, resulting in individual correlation maps between the experimental regressors and the physiological measures.Gianaros et al. (2005)showed that higher arterial blood pressure during the Stroop task was associated with greater activity in the pregenual and mid anterior cingulate cortex, the insula, and different regions of the medial prefrontal cortex.Causse et al. (2022)report positive blockwise HR modulations of the BOLD response in the left middle occipital gyrus, the right hippocampus, precuneus, and posterior cingulate cortex during a stressful n-back task.

In our study, we aim to weight the intraindividual stress dynamics by integrating the HR per experimental block as a marker of the peripheral stress response in an adapted version of the MIST. This is achieved by incorporating individual HR traces in the first-level models as a parametric modulator of the task regressor (mental calculations versus rest), which enables us to examine how the individual’s HR is linked with brain activity during periods of stress.

Since HR and HRV are anti-correlated (Kazmi et al., 2016, correlation coefficient in our data between HR and high-frequency power of HRV was r = -0.51, seeSupplementary Fig. 4), we first addressed HR per block to potentially capture the cardiovascular arousal state of each participant.

Here, we focus on three regions of interest (ROIs), including the bilateral amygdala, hippocampus, and insula since these regions have been repeatedly reported in meta-analyses and reviews on stress-imaging tasks (Berretz et al., 2021;Dedovic, D’Aguiar, & Pruessner, 2009;Kogler et al., 2015;Noack et al., 2019;Qiu et al., 2022). The amygdala and hippocampus are involved in affective processing (Buhle et al., 2014;Davis & Whalen, 2001;Lindquist et al., 2012;Seymour & Dolan, 2008), and the hippocampus is notably one of the key regions of HPA axis regulation (Herman et al., 2016,^2020^). Furthermore, meta-analyses consistently report the insula’s involvement in the stress response (Berretz et al., 2021;Kogler et al., 2015;Qiu et al., 2022).

We anticipated that the whole-brain analysis of the model incorporating blockwise individual HR would reveal statistical maps with bilateral clusters in these regions and that the ROI analysis would yield larger effect sizes compared with the control models due to combining an immediate physiological marker with the experimental manipulations in this model.

## 2 Materials and Methods

### 2.1 Participants

For the current analysis, 88 healthy participants of the ongoing transdiagnostic Biological Classification of Mental Disorders (BeCOME) study (Brückl et al., 2020) were analyzed (57 females, mean [sd] age 34.8 [11.7] years). The study is in accordance with the Declaration of Helsinki for human experimentation as amended at the General Assembly in October 2013 (World Medical Association, 2013) and was approved by the local ethics board (ClinicalTrials.gov: NCT03984084, reference number: 350-14). All participants gave written consent to participate after receiving full information about the study protocol. Participants were reimbursed after their participation. MRI suitability was determined before participants entered the scanner, and those who did not meet the MR-inclusion criteria were excluded. As outlined in the study design, none of the participants were taking psychopharmacological medication. For a detailed description of the study’s recruitment strategies as well as inclusion and exclusion criteria, we refer you to the overview given byBrückl et al. (2020).

For this work, participants recruited until November 2020 were analyzed. All participants were considered healthy and eligible for the study if they did not fulfill the criteria for any current full or subthreshold disorder according to the Munich-Composite International Diagnostic Interview (DIA-X/M-CIDI) (Wittchen & Pfister, 1997) conducted by trained study assistants one to two weeks prior to the stress test. The interview assesses multiple mental diagnoses according to DSM-IV together with information on onset, duration, and severity.

### 2.2 Experimental procedure

Participants performed a stress task based on the MIST (Dedovic et al., 2005), which was adapted by splitting the ‘control’ condition into two phases (PreStress and PostStress phases without negative social feedback) and by further subdividing each task phase into five interleaved blocks of active task performance and rest blocks (Elbau et al., 2018;Kühnel et al., 2020,2022). The task has been validated for its effectiveness in eliciting a cardiovascular, endocrine, and behavioral stress response (Elbau et al., 2018). Here, we use the version specifically adapted for clinical settings (Brückl et al., 2020;Kühnel et al., 2020,2022).

In three task phases (*PreStress, Stress, PostStress*), totaling approximately 25 min, participants were asked to solve mental calculations. Each task phase is subdivided into five blocks (60 s active task block followed by 40 s rest block). It should be added that one active block contained about 5-6 cycles of mental calculation presentations, calculating, answer submission, and visual feedback. Shortly before the*Stress*phase, participants were informed by the experimenter that their performance would now be monitored and recorded. Only during the second phase, the psychosocial*Stress*phase, additional negative feedback and time pressure were applied, while these stressors were absent in*PreStress*and*PostStress*phases. The mental calculations had three levels of difficulty that were balanced between the three task phases. During the*Stress*phase, the time limit per calculation was set to 4.5 s and further adjusted (shortened) if participants performed well. The task is depicted inFigure 2.

**Fig. 2.**
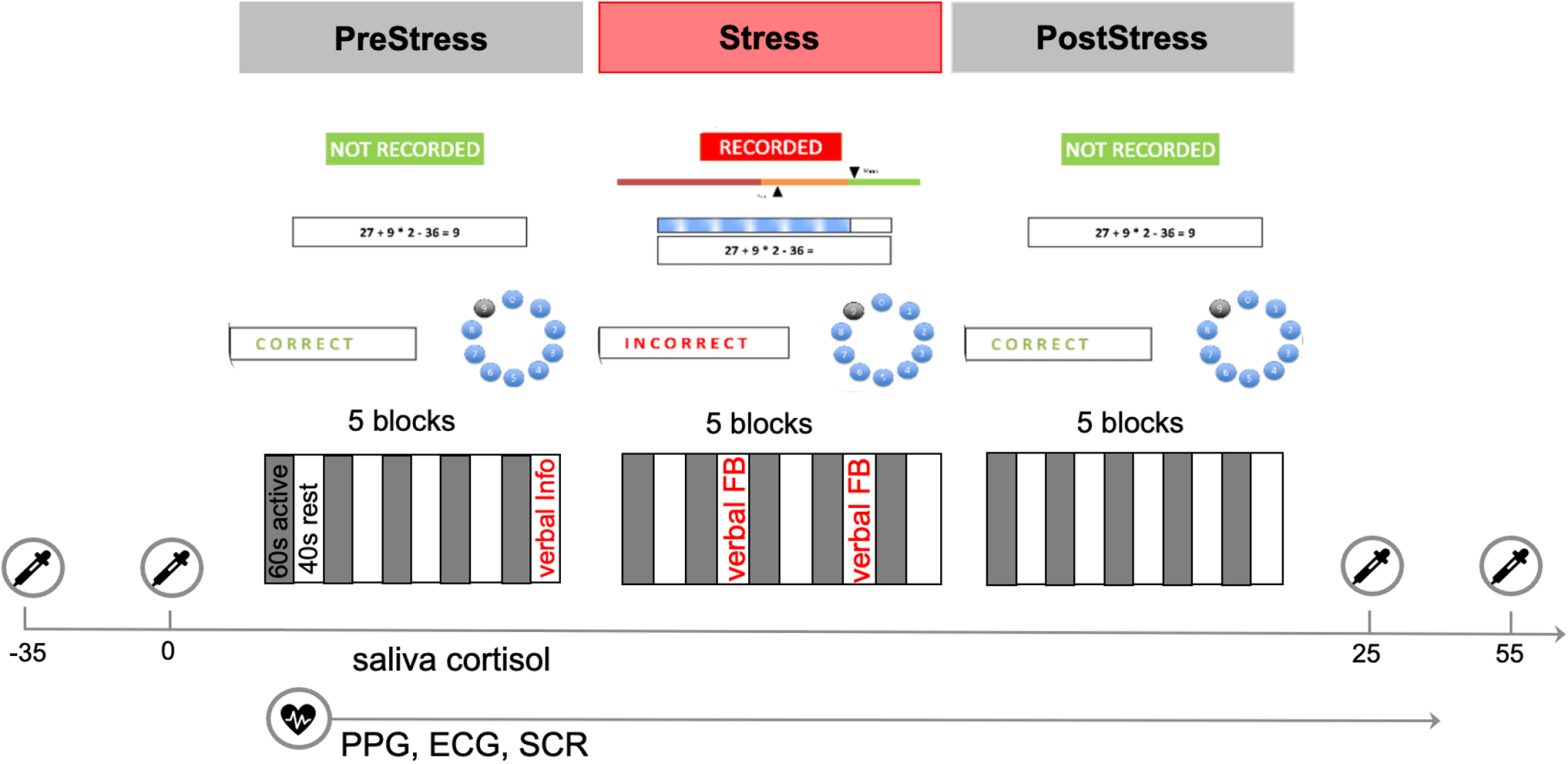
Schematic illustration of the task. The task was imbedded into the second day of the beCOME study and consisted of three different phases (PreStress, Stress, and PostStress) with interleaved blocks of active calculations (60 s) and rest (40 s). East task phase consisted of 5 blocks; 3 rest blocks verbal instructions or negative verbal feedback were presented. PPG, ECG, and skin conductance were measured simultaneously; saliva cortisol was collected at four different times (in minutes) throughout the task. Figure adapted fromKühnel et al. (2020)usingBioRender.com.

During the entire task, HR was measured using photoplethysmography (PPG) with an MR-compatible pulse oximeter (Nonin 8600FO, Nonin Medical Inc., Plymouth MN, USA) attached to the left ring finger. For the PPG recordings, the analog PPG output was forwarded to the multi-channel Brain Vision Recorder software (version 1.0,https://www.brainproducts.com/support-resources/brainvision-core-data-format-1-0/) with a sampling rate of 5 kHz. Additionally, skin conductance responses and electrocardiography were measured. A total number of four saliva cortisol samples were taken before and after the task. Additionally, self-ratings of the participants’ stress-levels were obtained before and after the task. The subjective and endocrine responses to the task have been described byKühnel et al. (2020,2022).

### 2.3 Assessment of subjective experiences

Subjective experiences of stress were measured before, immediately after, and 30 min after the task using the BSKE (Befindlichkeitsskalierung durch Kategorien und Eigenschaftswörter; “mental state scale by categories and adjectives”) (Janke, 1994). Participants were asked to state their current feeling on a 6-point-Likert scale from “not at all” to “very strongly”. Two sum scores were calculated: one on positive valence and one on negative valence. For further details seeKühnel et al. (2020,2022).

### 2.4 MRI data acquisition and preprocessing

MRI data were acquired on a 3 Tesla scanner (General Electric, Discovery MR750, Milwaukee, WI, USA) with a 32-channel head coil. Whole-brain data were collected covering the whole brain in interleaved 40 slices (AC-PC orientation of the slices, 3 mm slice thickness, 0.5 mm slice gap, resulting voxel size 3.5 × 3.5 × 3 mm^3^, 64 × 64 matrix, field of view 20 × 20 cm^2^). 755 volumes using a T2*-weighted echo-planar imaging sequence (time of repetition [TR] 2 s, time of echo [TE] 30 ms) were recorded of which the first five volumes were discarded to account for T1-equilibrium.

After rigid-body realignment, volumes with motion-related outlier characteristics were identified using FSL root mean square of intensity difference (*dvars*) as well as frame-wise displacement differences (*fdrms*) (Power et al., 2012;Smyser et al., 2011). Average*fdrms*values were calculated and compared to the critical exclusion threshold of 1.5 mm as in our previous stress task analyses (Kühnel et al., 2022). No participants were excluded due to excessive motion. Thereafter, preprocessing was performed in Matlab 2018a and Statistical Parametric Mapping (SPM12, Wellcome Centre for Human Neuroimaging, London, UK,https://www.fil.ion.ucl.ac.uk/spm/software/spm12/) and consisted of nine subsequent steps: (1) To account for motion, rigid-body alignment using the first image of the scan as a reference was performed. (2) The resulting volumes were corrected for slice timing differences. (3) The contrast-rich T2-weighted EPI acquired immediately before volumes (interleaved bottom-up approach, 2D spin echo DTI, 128 x 128 matrix, field of view 25.6 x 25.6 cm^2^, TR 8 s, TE 60.9 ms) was co-registered to the average regular EPI image. (4) That same T2 image was segmented (Ashburner & Friston, 2005). (5) Grey and white matter segments were forwarded to the DARTEL spatial normalization using default IXI templates (sic cycles), resulting in flow fields. (6) The volumes, as well as cerebrospinal fluid (CSF) and white matter (WM) maps were normalized to the Montreal Neurological Institute (MNI) space using these flow fields (7) and interpolated to a voxel resolution of 2 × 2 × 2 mm^3^. (8) The resulting volumes were residualized against the six rigid-body motion parameters, differential motion, and*dvars*derived motion outliers and linearly detrended in a general linear model (GLM). On the residualized volumes from this first step, the first five principal components of the individual CSF and WM compartments were extracted and used as regressors of no interest in the final first-level models, following the CompCor concept (Behzadi et al., 2007). (9) Spatial smoothing (3D Gaussian kernel, full width at half maximum 6 × 6 × 6 mm^3^) was performed on the residualized volumes, resulting from step (8).

### 2.5 Data analysis

#### 2.5.1 Calculation of individual HR and analysis of behavioral data

PPG data were analyzed similarly as described inElbau et al. (2018)andKühnel et al. (2020,2022). Raw PPG data were preprocessed in Matlab R2020b with the PhysioNet-Cardiovascular-Signal toolbox (https://github.com/cliffordlab/PhysioNet-Cardiovascular-Signal-Toolbox). Data were first resampled to 100 Hz, and Brain Vision markers were used to identify the beginning and end of the task. Automated peak detection was performed with the*Analyze_ABP_PPG_Waveforms*function as incorporated in the PhysioNet-Cardiovascular-Signal toolbox. The resulting peaks were marked and plotted on the resampled signal for each participant. Each plot was inspected visually and rated on a 3-point rating scale whereby lower values reflected a better peak detection. The criteria for satisfactory peak detection included assessing the frequency of missed peaks and the accuracy in distinguishing the actual peak from deviations immediately preceding or following it. Participants with PPG traces throughout the entire task with a visual rating of >2 were considered of insufficient quality and excluded (N = 5). The final sample consisted of 83 participants (55 females, mean [sd] age 35.1 [11.1] years). For these participants, the peak-to-peak intervals were calculated and forwarded to the R-based RHRV package (Rodríguez-Liñares et al., 2008), in which we obtained HR and—as a control—high-frequency heart rate variability (HF-HRV, 0.15-0.40 Hz ). HF-HRV reflects a compound marker of respiration frequency and the shift in vago-sympathetic balance under stress (Castaldo et al., 2015) and is highly correlated with the root-mean-square of successive differences (RMSSD) between normal heartbeats, with correlations of up to 0.93 (Kleiger et al., 2005;Quintana, Elstad, et al., 2016;Shaffer & Ginsberg, 2017;Thomas et al., 2019). HR, in contrast, is less influenced by respiratory changes (Shaffer & Ginsberg, 2017). To filter out artifacts of unrealistic fast changes in HR (e.g., the HR cannot increase from 50 to 150 in 5 s), an average HR of 50 heartbeats was taken and a 12% increase or decrease allowed for the successive heartbeat (default values recommended by the package authors). For the HF-HRV calculation, a window size of 300 s with shifts of 30 s was taken (as recommended by the package authors). This results in the estimation of HF-HRV for every 30 s in the recording employing a Fast Fourier transformation approach. Then, the data were averaged per participant and active calculation block (see section 2.4.2) and volume (see also section 2.4.2) to obtain HR and HF-HRV.

To measure performance, the accuracy of the participant’s responses to the mental calculation was calculated for each of the three task phases. The accuracy measures and the mean HR were compared between the task phases using repeated-measures ANOVA.

#### 2.5.2 First- and second-level fMRI analysis

**Fig. 3.**
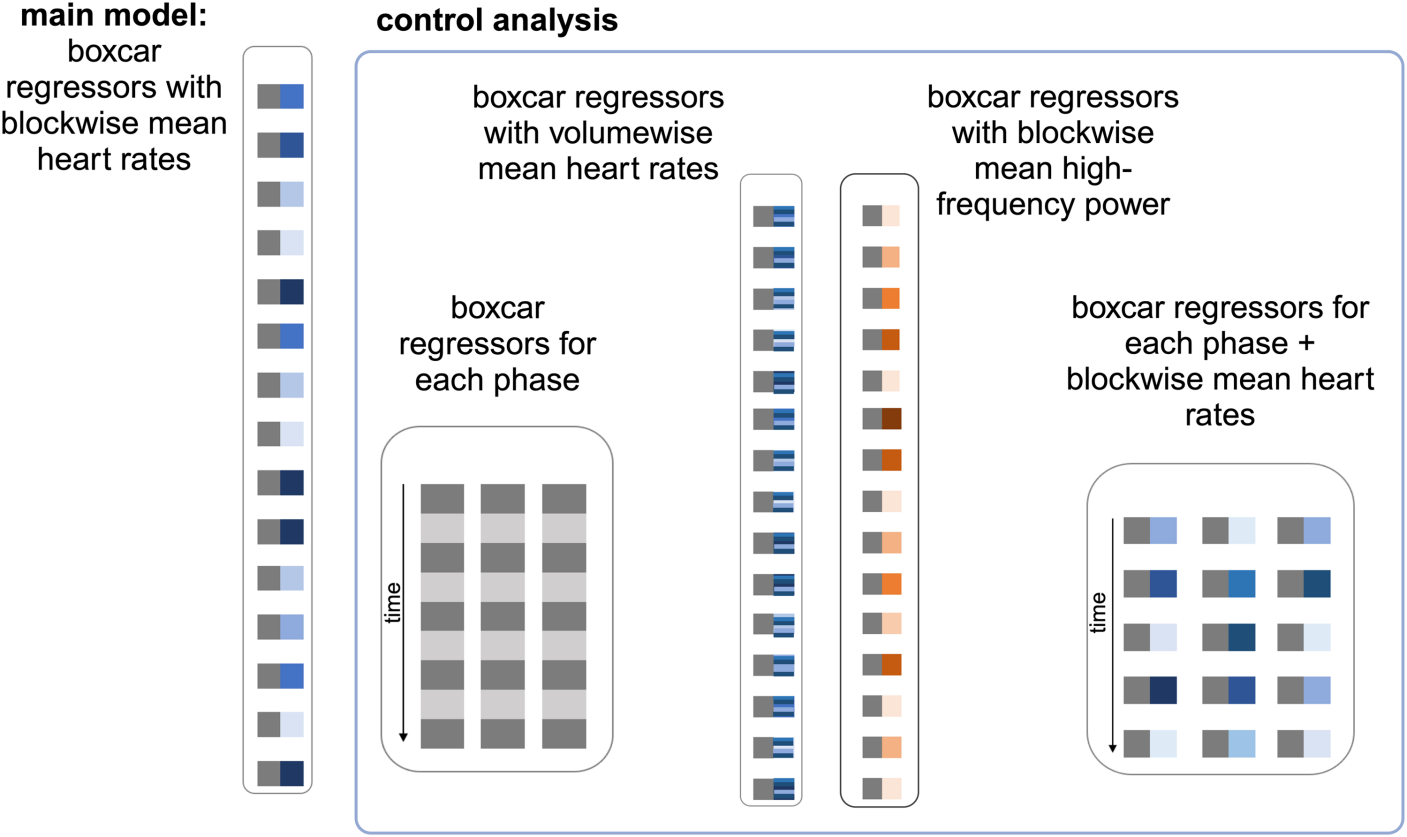
Regressors for the model with blockwise pulse rates as parametric modulator and for the control analysis. Dark grey boxes represent box-car regressors for active task blocks (either regardless of the phase or for task phases sepereted), and blue/orange boxes represent either blockwise or volumewise heart rates/high-frequency power as a parametric modulator of the box-car regressors. This figure has been modified withBioRender.com.

To examine the usefulness of weighting the experimental regressors by individual HR as a marker of the stress response, we computed first-level GLMs with a single regressor capturing all fifteen active blocks (regardless of the stress phase) with individual mean HR per active block added as parametric modulator (Fig. 3). In addition to its respective main regressor, each first-level model contained the first five principal components from the time series of voxels within individual WM and CSF masks (as gained from thresholded [0.99 and 0.95, respectively] tissue probability maps). The high-pass filter was set to 256 s. Random effect second-level analysis for the model was composed of a one-sample t-test [+1], [-1] of the parametric modulator forwarded to the group level.

In addition, for comparison with other fMRI studies on the imaging stress task, we computed a task-regressor based model containing one boxcar regressor for the five calculation blocks per task phase and with the differential first-level t-contrast between*Stress*and aggregated*PreStress*and*PostStress*(contrast vector [-1 +2 -1]) forwarded to the group level (Supplementary Fig. 2).

As control analyses, we computed three additional models to explore volumewise temporal changes, to examine the effect of the experimental phase (phasewise HR-model with blockwise mean HR), and to explore the effect of using blockwise HRV-HF in the Control Analysis.

Second-level group contrasts were thresholded at the voxel level, taking into account multiple testing corrections with family-wise error correction. Voxels with p_voxel.FWE_< 0.05 in clusters >25 voxels are reported. For exploratory purposes, an uncorrected threshold was applied at the voxel level (p_voxel_< 0.001, cluster threshold = 25 voxels,Supplementary Fig. 3) for the task-regressor based model, to ensure we did not miss any subthreshold activity in relevant regions.

To test our hypothesis that using blockwise individual HR as a parametric modulator would result in stronger effect sizes in the specified regions (amygdala, hippocampus, and insula), we computed Cohen’s D effect sizes for the main model (blockwise HR-model) to form the region’s beta coefficients compared to the control analyses for the ROIs. The reported effect sizes represent the differences in mean beta coefficients in the respective regions in pooled standard deviations.

#### 2.5.3 Regional BOLD timecourse analysis

The first principal component of three ROIs, which showed significant correlation with the HR in our main model, was extracted from the preprocessed time-series. ROIs were the bilateral amygdala, bilateral hippocampus, and bilateral insula taken fromRolls et al. (2020). The average timecourse across all participants and its 95%-confidence intervals per region was calculated. Further, to derive task phase specific timecourses, averages across the five respective active and rest blocks per task phase were calculated.

#### 2.5.4 Association of subjective measures and HR

To explore which subjective measures of the BSKE correlate with which physiological values of the stress response (HR), we calculated the correlations between these measures in a post hoc analysis. For HR, three values were calculated: the mean HR increase from the PreStress to Stress phase (upswing),the mean decrease from the Stress to PostStress phase (downswing), as well as the proportional downswing (normalized by the upswing) for each participant. These values were probed for correlations with changes in the BSKE scales of positive and negative emotions as well as with the changes of specific items from pre- to post-task. Correction for multiple comparisons was done by first calculating the number of effective tests (to account for correlations between the subjective rating scales and items) using the*meff*function of the R-package*poolr*and then dividing an alpha-error rate of 5% by that number (resulting in a p-threshold of 0.01).

## 3 Results

### 3.1 Manipulation check

Subjective experiences were significantly affected by the stress task. The intensity of self-reported negative affect was higher after the task and recovered 30 min after the end of the experiment (F_(167.1, 1.7)_=54.1, p < 0.0001, Mean_before_[sd] = 14.3[4.5], Mean_rightAfter_[sd] = 21.9[7.8], Mean_30minAfter_[sd] = 13.4[4.4],Supplementary Fig. 1), while self-reported positive affect decreased throughout the task and did not recover to baseline 30 min after the end of the experiment (F_(166,2)_=7.6, p < 0.0001, Mean_before_[sd] = 20.7[3.5], Mean_rightAfter_[sd]=18.5[3.8], Mean_30minAfter_[sd] = 19.3[4.1],Supplementary Fig. 1). This shows that participants experienced stronger self-reported negative and weaker self-reported positive affect due to the task.

### 3.2 HR and behavioral performance

**Fig. 4.**
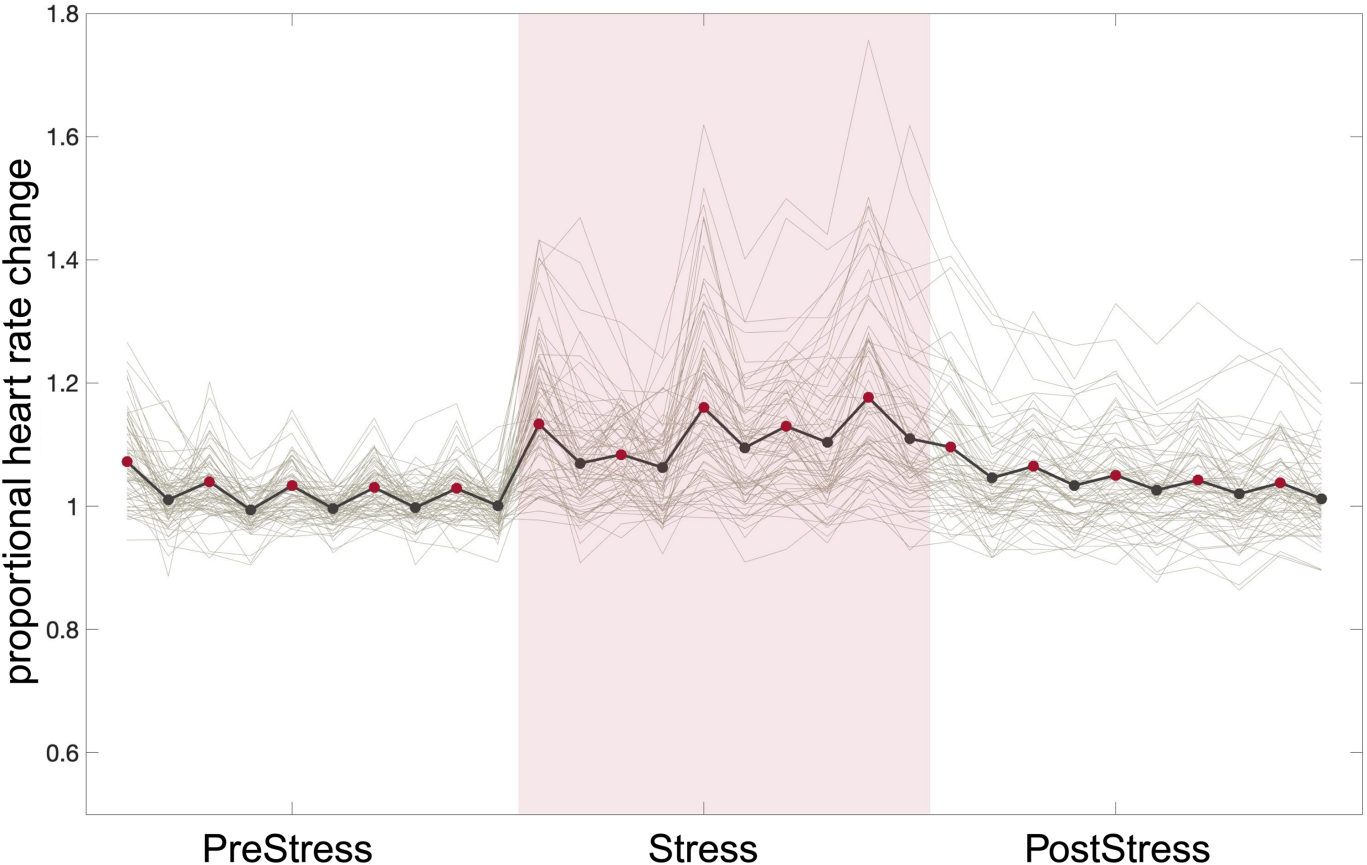
Heart rate traces across the task. Solid black line shows the mean heart rate changes across the task; red dots= active calculation blocks, black dots= rest blocks; grey lines show individual heart rates; and all data have been normalized to the mean heart rate during rest in the PreStress phase. Thus, values >1 represent heart rate increases relative to the rest in the PreStress phase and <1 vice versa. This figure has been modified withBioRender.com.

HR was significantly influenced by the task phase (F_(141.8, 1.7)_= 54.7, p = 0.016), with higher HR (mean[sd] = 81.4 bpm [22.7 bpm]) during the active calculation blocks during*Stress*compared to the*PreStress*(mean[sd] = 74.6 bpm [23.4 bpm]) and*PostStress*condition (mean[sd] = 75.9 bpm [23 bpm];Fig. 4).

Regarding the behavioral performance, the accuracy was lower during the*Stress*phase (mean accuracy[sd] = 41% [19%]; F_(166,2)_= 161.9, p < 0.0001) compared to the other phases (*PreStress*: mean accuracy [sd] = 58% [22%];*PostStress*: mean accuracy [sd] = 59% [18%];Supplementary Fig. 1).

### 3.3 Correlation of HR changes and subjective ratings

**Fig. 5.**
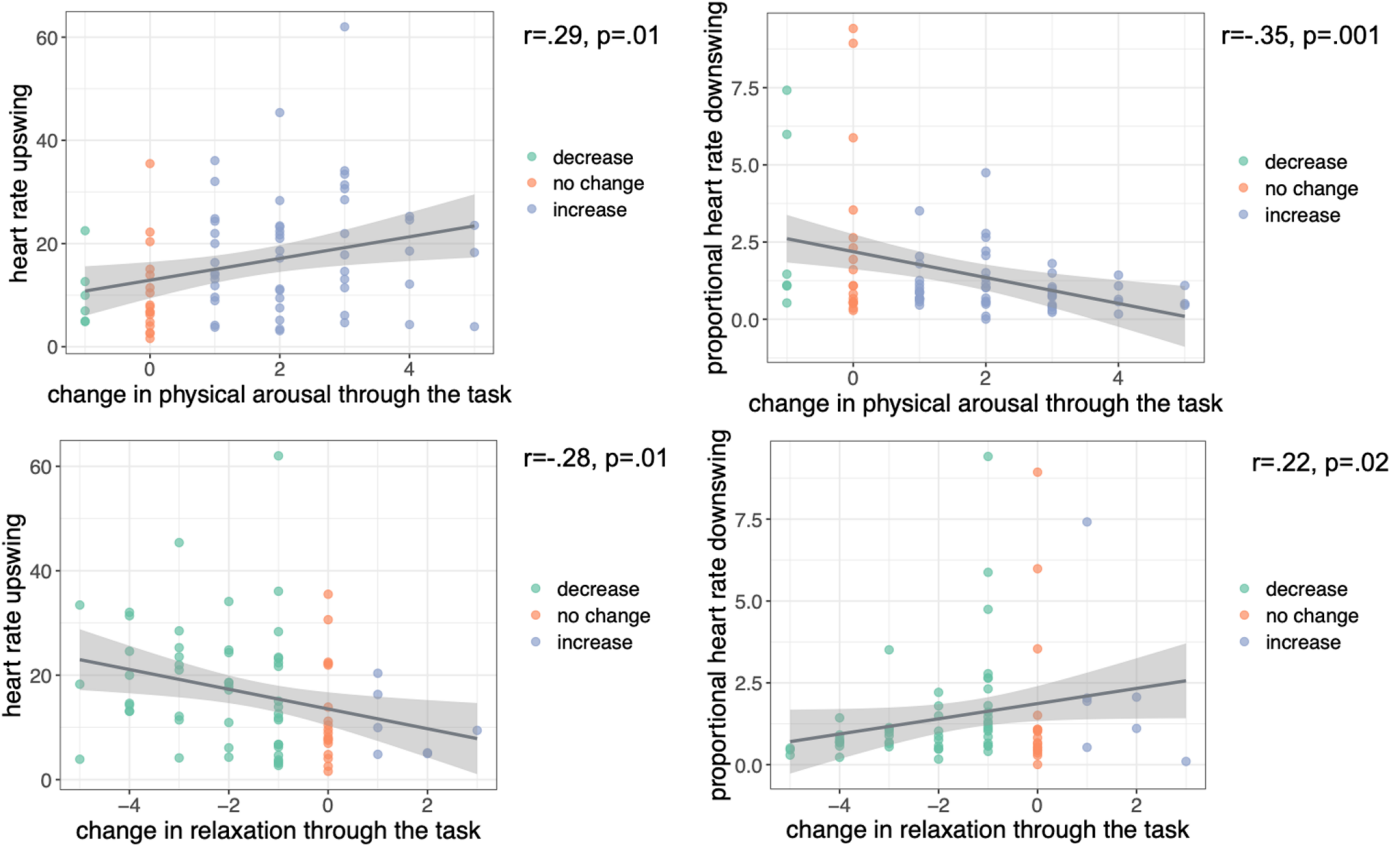
Correlation of the heart rate up- and downswings with changes in self-reported physical arousal and relaxation from the BSKE. Left: correlations with mean heart rate upswing from the PreStress to Stress phase with changes in physical arousal and relaxation; Right: same but with proportional heart rate downswing (normalized to the participant’s upswing); r = correlation coefficient, p = uncorrected p-value (after correcting for multiple comparisons with the number of effective tests calculated with the R-based poolr package the p-threshold for significance was 0.01). This figure has been modified withBioRender.com.

HR upswing was positively correlated with the self-reported change in physical arousal (r = 0.29, r = 0.01) and negatively correlated with relaxation (r = -0.28, p < 0.01) before versus after completing the task. Proportional HR downregulation was negatively correlated with the self-reported change in physical arousal (r = -0.35, p = 0.001) but positively correlated with the self-reported change in relaxation through the task. The correlation with self-reported change in relaxation was not roboust to correction for multiple comparisons (r = 0.22, p = 0.02,Fig. 5). The correlations with the sum scores of positive and negative emotions were non significant, which indicates a certain specificity for HR to be associated with self-reported physical arousal states.

### 3.4 HR is correlated with insula and anticorrelated with hippocampus, amygdale, and default mode network activity

**Fig. 6.**
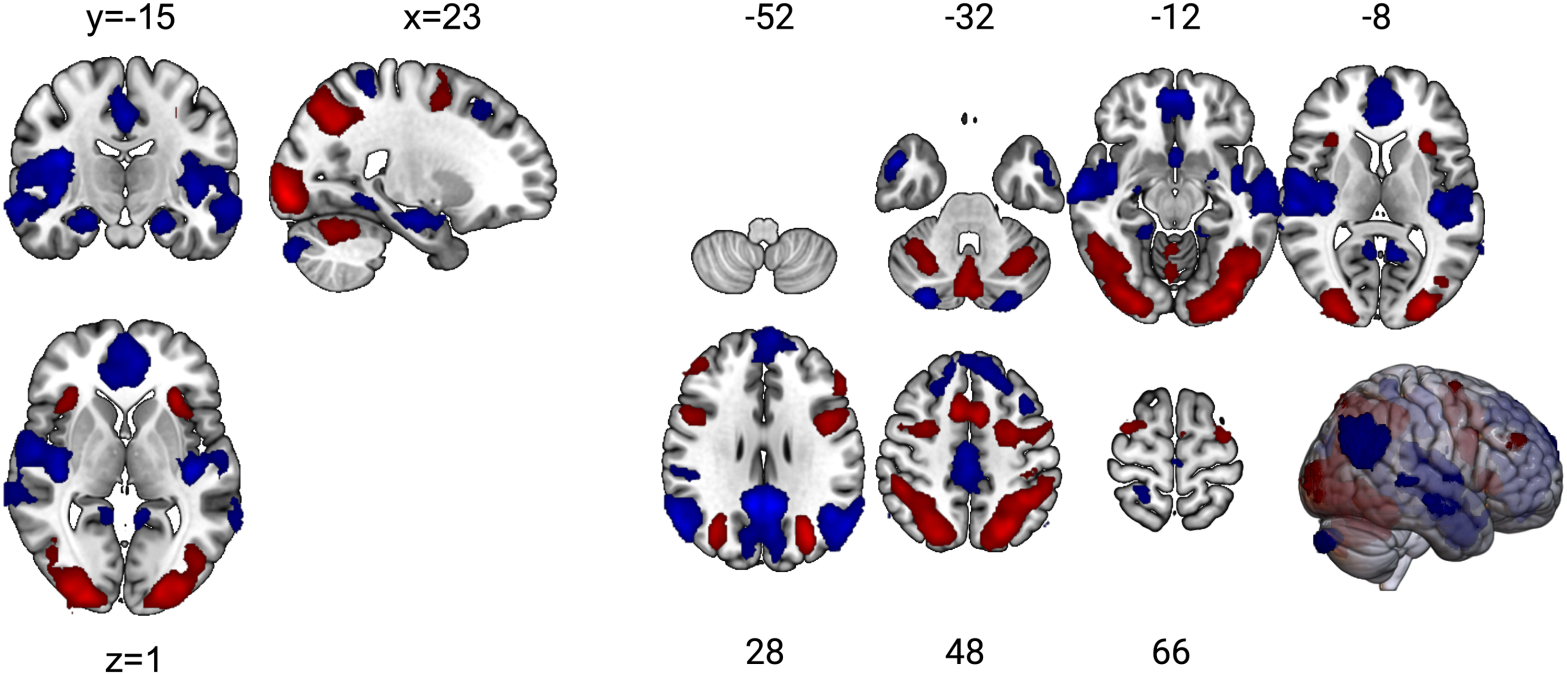
Main results of the model incorporating individual mean heart rates. Depicted are neural correlates with a parametric modulator across all 15 active blocks of individual mean heart rates. The map is thresholded at p_voxel.FWE_<0.05 and cluster extent >25. Red = positive correlation with heart rate. Blue = negative correlation. This figure has been modified withBioRender.com.

The Model with individual HR as a parametric modulator to all active blocks (irrespective of task phase,Fig. 3) revealed positive correlations in the bilateral insula, cerebellar vermis, bilateral middle frontal gyri, angular gyrus, inferior and superior parietal lobules, as well as bilateral supplementary motor area, bilateral precentral gyrus, the right inferior temporal gyrus, and bilateral fusiform gyrus. Negative correlations were observed mainly in the superior frontal gyrus and medial orbitofrontal gyrus, bilateral precuneus, and parts of the rostral anterior and posterior cingulate cortex, which all typically represent the task-negative default mode network (DMN) (Buckner & Vincent, 2007). Negative correlations were further observed in the bilateral middle frontal gyrus, temporal gyri, hippocampus, parahippocampal gyrus, amygdale, and putamen (Fig. 6,Table 1).

**Table 1.** Clustertable.

Model and contrast	Peak coordinates			Label	k	% of k	p	t
	x	y	z					
Blockwise HR model: +1	22	-92	-4	Left middle occipital lobe	22737	11.17	0.000	19.25
				Left inferior parietal lobe		8.64		
				Right cerebrum		6.42		
	-6	6	52	Left precentral gyrus	6698	22.10	0.000	17.03
				Left supplementary motor area		10.50		
				Right precentral gyrus		8.72		
				Right superior frontal gyrus		7.06		
				Left superior frontal gyrus		6.57		
				Left inferior frontal gyrus		6.29		
				Right supplementary motor area		5.99		
	34	20	4	Right insula	313	69.97	0.000	10.63
	42	44	30	Middle frontal gyrus	488	75.82	0.000	10.12
				Right inferior frontal gyrus, pars triangularis		21.11		
	6	-28	-6	Brain stem	111	40.4	0.000	9.39
				Right ventral DC		13.3		
	-26	36	-28	Left temporal pole	1285	96.6	0.000	7.36
	-12	-16	-6	Thal_VL_L	83	43.37	0.000	8.4
				Thal_MDl_L		22.89		
				Thal_IL_L		10.84		
				Thal_PuA_L		8.43		
	-18	-42	-44	Left lobule X of cerebellar hemisphere	27	44.44	0.000	7.58
				Left lobule IX of cerebellar hemisphere		43.9		
	8	72	-12	Right superior frontal gyrus, medial orbital	38	81.42	0.003	6.53
Blockwise HR model: -1	6	-52	30	Left middle temporal lobe	34991	6.36	0.000	18.92
				left superior frontal gyrus, medial orbital		5.61		
				Right superior temporal lobe		5.38		
	30	-82	-32	Right crus II of cerebellar hemisphere	678	55.60	0.000	13.22
				Right crus I of cerebellar hemisphere		28.32		
	-20	-88	-38	left crus II of cerebellar hemisphere	508		0.000	12.2
	22	-42	60	Right postcentral gyrus	877	54.39	0.000	10.61
				Right precentral gyrus		29.65		
				Right superior parietal lobe		9.46		
	-24	-44	60	Left superior parietal lobe	103	56.31	0.000	9.11
	6	-52	-46	Right cerebelum	41	100	0.000	8.15
	36	34	-12	Right Inferior frontal gyrus, orbital part	57	78.95	0.000	7.83
	-18	-34	62	Left postcentral gyrus	27	59.26	0.001	6.8
				Left paracentral lobe		25.93		

Model name, coordinates of the peak voxel, clustersize (k), region labels as revealed by the AAL3 toolbox including % voxel (% of k) of the cluster labeled with the respective name, p- and t-value, Note: only clusters of at least 5% of the voxels are shown.

The contrast [-1 +2 -1] of the task-regressor based model revealed stronger activation during the*Stress*phase in bilateral occipital and temporal areas, including the lingual gyri, the calcarine sulcus, and the left fusiform area. Additionally, the left parahippocampal gyrus, right hypothalamus, and right precuneus were stronger activated during*Stress*compared to the two other phases. Deactivation during*Stress*was observed in the bilateral middle temporal gyri, left superior temporal gyrus, right insula, as well as the right pre- and postcentral gyri (Supplementary Fig. 2,Supplementary Table 1). At a lower threshold (uncorrected p_voxel_< 0.001,*k*> 25 voxels,Supplementary Fig. 3), additional*Stress*associated activations emerged in the brainstem (potentially including the locus coeruleus) and the bilateral thalamus and middle cingulate cortex. Additional deactivations emerged in the left superior frontal gyrus, left supplementary motor area, left precentral gyrus, as well as bilateral superior temporal gyrus.

The control analysis with volumewise HR as a parametric modulator revealed a statistical map similar to the blockwise HR model but without positive correlations with the insula. Furthermore, activity in the bilateral supramarginal gyri and clusters in the occipital cortex were positively correlated with volumewise HR (Supplementary Fig. 2,Supplementary Table 1).

The differential contrast of the phasewise model with mean HR is reported in the Supplementary Results and revealed differential effects in the left insula with a weaker correlation to HR during the*Stress*compared to the combined*Pre*and*PostStress*phases.

The HF-HRV model revealed a partially reversed map compared with the main model. For example, the amygdala, hippocampus, and orbitofrontal cortex were positively correlated with HF-HRV. This is likely due to the negative correlation between HR and HRV-HF (r = -0.51, p < 0.001,Supplementary Fig. 4).

For more details on the methods and results of this control analysis, we refer to the Supplementary Material (Control Analysis, Supplementary Results,Supplementary Fig. 2andSupplementary Table 1).

A comparison of the effect sizes for the main model with those of the control models showed that the effect sizes for the bilateral amygdala, hippocampus, and insula were larger in the main model compared to the others (Table 2) with beta-values being significantly different for the comparison of the main model and the task-regressor based, HRV-HF and HR-phasewise models for the amygdala and hippocampus. Only the beta values from the volumewise model versus the main model for the amygdala and hippocampus were non-significant. The same was true for the comparison of the main model and the phasewise model with mean HR as parametric modulator. All other effect sizes were significant, which suggests that using HR as a parametric modulator provides a more sensitive approach to detect activity in these regions.

**Table 2.** Effect sizes for the main model compared to the control models.

	Main model vs. task-regressor based model	Main model vs. volumewise model	Main model vs. HF-HRV-model	Main model vs. HR-phasewise model
Amygdala	-0.354***	-0.189 ^n.s.^	-1.001***	-0.481***
Hippocampus	-0.247**	-0.08 ^n.s.^	-0.954***	-0.362***
Insula	0.169*	0.293***	0.217*	0.169 ^n.s.^

Bilateral ROIs were combined for this analysis.

n.s. = non-significant, *p < 0.05, **p < 0.01, ***p < 0.001

### 3.5 Timecourse analysis

**Fig. 7.**
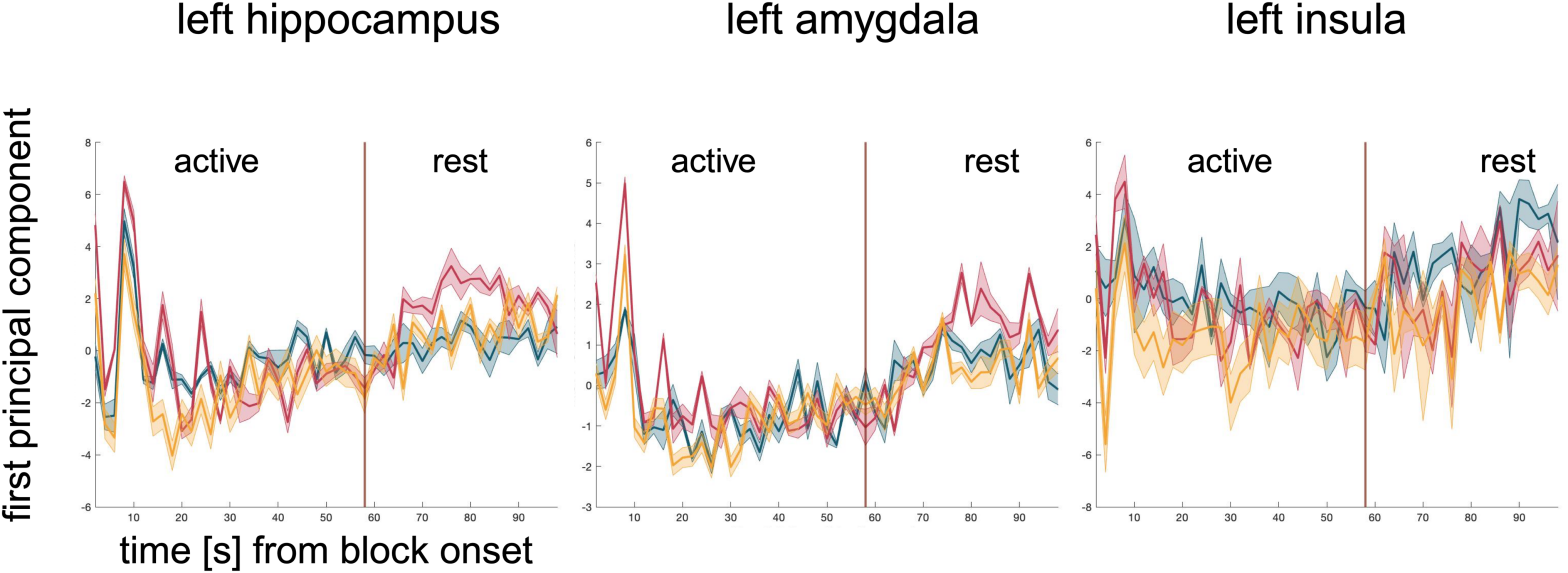
Mean z-transformed regional time-courses across blocks per task phase. Depicted are Mean z—transformed first principal components for left hippocampus, left amygdale, and left insula for all three task phases (blue =*PreStress*, red =*Stress*, yellow =*PostStress*) averaged across all active and rest blocks of each respective task phase and 95% confidence intervals (shaded areas). Similar results were obtained also for the right hippocampus, right amygdala, right insula (not shown). This figure has been modified withBioRender.com.

To illustrate activity changes across the task, the first principal component of the three different ROIs was visually compared (Fig. 7): In the bilateral amygdala and the hippocampus, we observed stronger activation during the active blocks of the*Stress*phase as compared to the*PreStress*and*PostStress*phases. This manifested in non-overlapping 95%-confidence intervals from second 6 to 10 for the amygdala and 10 to 12 s for the hippocampus. In the*Stress phase*, increased activity at the block onsets was observed in the insula as well, although to a lesser extent compared to the amygdala and hippocampus (Fig. 7).

For all three ROIs, a pronounced peak in the activity was observed at the beginning of all active blocks (Fig. 7), raising the methodological concern that the results could have been confounded by block-onset related artifacts due to co-occurring physiological response (for example, blood pressure, perfusion or motion artifacts). Therefore, we modified the blockwise HR model (parametric modulation of HR per block) by excluding the first 10 s, and in another control analysis, the first 20 s, into a separate regressor of no interest. Although some clusters of activity changed slightly, the correlations with the bilateral hippocampus, amygdale, and insula of the model remained (Supplementary Fig. 2,Supplementary Table 2).

## 4 Discussion

In this study, we investigated the utility of incorporating individual HR traces for the detection of stress-related brain regions during the performance of an acute psychosocial stress task. Our results demonstrate that (1) integrating individual HR traces as a guide for fMRI analysis aids in sensitizing the analysis towards brain regions expected to be involved in regulating the stress response; (2) the experimental task-regressor model based on the calculation block design detected the hypothalamus but no task phase effect in the amygdala, hippocampus, or insula; (3) specifically, the amygdala and the hippocampus increased their activity immediately at the beginning of each mental calculation block onset and showed higher activity in general during the rest periods of the*Stress*phase compared to the two other phases; and (4) that the effect sizes of the model with blockwise HR as a parametric modulator showed greater effects in the amygdala, hippocampus, and insula compared to most of the control analyses.

In the present study, we used individual mean blockwise HR to index the dynamics of the stress response. The peripheral stress system represents the first, rapid-response layer, and in this context, we identified robust clusters of activity in the bilateral insula, amygdala, and hippocampus, with the parametric modulation of blockwise HR, which is anticorrelated with heart rate variability (Kazmi et al., 2016) (we observed an anticorrelation of r = -0.51 in our blockwise approach, seeSupplementary Fig. 4). In the control analysis with HF-HRV as parametric modulation, we also showed that this anticorrelation of HR and HF-HRV resulted in a partially reversed map. However, the comparison of the effect sizes (Table 2) in the three ROIs shows a clear advantage of the blockwise HR model compared to most control models.

In the present study, HR up- and downswings between the task phases were specifically correlated with self-reported items that reflect a participant’s physiological arousal (or relaxation as the opposite item). Thus, the perception of one’s arousal state and the cardiovascular arousal state align and make HR a suitable modulator that captures the individual stress dynamics while being correlated with self-reported arousal. The incorporation of blockwise mean HR combines the experimental stressors (task regressor) with the individual dynamic “stress wave” and thus provides a useful and robust complementation of the task-regressor based analysis.

The anterior hippocampus has been shown to play a critical role in regulating the stress response in various fMRI stress paradigms (Dedovic, D’Aguiar, & Pruessner, 2009;Khalili-Mahani et al., 2010;Noack et al., 2019) and stronger functional connectivity between the hippocampus, amygdala, insula, ventrolateral PFC, anterior cingulate cortex, and hypothalamus has been reported in participants with lower compared to higher resting HR (de la Cruz et al., 2019).Eisenbarth et al. (2016)showed that the hippocampus has a negative weight when predicting HR in the context of social evaluative threat. We observed a clear anti-correlation between activity in the hippocampus and HR variations across blocks. Deactivation is usually interpreted to accompany the initiation of a stress response that—if prolonged—would continue to involve the HPA-axis through disinhibition of the continuously suppressing gamma-aminobutyric acid (GABA) input from the hippocampus (Dedovic, D’Aguiar, & Pruessner, 2009;Herman et al., 2016). This interpretation is further supported by the finding of a linear relationship between hippocampal deactivation and cortisol increase (Pruessner et al., 2008) as well as with stress exposure time (Henze et al., 2020).

The direct GABA-ergic projections from the ventral subiculum to the paraventricular nucleus (PVN) of the hypothalamus may not only be interpreted as HPA-axis initiation but also as disinhibition of the PVN and thereby autonomic response regulation. The PVN is a central hub for efferent autonomic regulation (Ferguson et al., 2008;Lamotte et al., 2021). The disinhibition is further supported by a study reporting a negative correlation between hippocampal BOLD and instantaneous indicators of cardiovascular complexity at rest (Valenza et al., 2020) and hippocampal activity reduction in response to parasympathetic perturbation employing non-invasive carotid stimulation (Makovac et al., 2015). However, since most studies are correlative and show similar correlations with the amygdala (Makovac et al., 2015; our own analyis) and given the anatomical evidence of a direct, fast stimulation of the sympathetic nervous system from the amygdala reaching the hypothalamus through the stria terminalis during the processing of stressful or fear-related stimuli (Kruger et al., 2015;Somerville et al., 2010), it is challenging to dissect the degree of modulation exerted solely by the hippocampus.

Hippocampal deactivation also occurs in sync with and as a putative part of the default mode (‘task-negative network’) deactivation during goal-directed cognitive tasks (Salgado-Pineda et al., 2017;Spreng, 2012). For this, task difficulty is often reported to predict the degree of deactivation (Cousijn et al., 2012). In this line of thinking, considering the observed inter-individual variations across all three phases, the fact that the HR weighting reveals a hippocampal involvement is consistent with the notion that HR tracks the ‘subjectively experienced’ and physiologically expressed task difficulty.

The negative correlation between amygdala activity and HR is in line with decreased amygdala activity during psychological stress from previous reports (Berretz et al., 2021;Henze et al., 2020;Noack et al., 2019;Wager et al., 2009). Moreover,Ginty et al. (2013)reported greater amygdala deactivation in response to another task (here: multisource inference task) specifically in a group of participants with blunted cardiovascular responses to a Paced Auditory Serial Arithmetic Test. Similarly, in a meta-analysis byBeissner et al. (2013), autonomic reactivity under cognitive and affective challenges showed that the amygdala was involved in both sympathetic and parasympathetic regulation. The amygdala is also involved in the initiation of the HPA axis through downregulation of GABAergic input to the PVN (Herman et al., 2016). However, in addition to serving as a mechanism for initiating stress responses, these results could further be interpreted as correlates of downregulation:Berretz et al. (2021)assert that decreased amygdala activity during the stressor may arise from the participant’s effort to (down)regulate the stress-induced emotion to enhance performance. Furthermore, in a study comparing a group receiving neurofeedback based on their amygdala activity with a control group employing an emotional coping strategy during exposure to emotional pictures, the neurofeedback group showed greater amygdala downregulation (Herwig et al., 2019). This is in line with a meta-analysis fromBuhle et al. (2014), who reported an attenuation in bilateral amygdala activity as a correlate of cognitive control to negative emotions.Makovac et al. (2015)used an emotional intensity paradigm, in which participants perceived emotional faces of different valences and rated the subjectively evoked affect. The experimenters perturbed the parasympathetic drive during stimulus presentation and subjective ratings with non-invasive carotid stimulation in half of the trials, which resulted in decreased activity in the amygdala, insula, hippocampus, thalamus, and temporal fusiform areas compared with the control condition. Interestingly, participants who rated the emotional stimuli most negatively exhibited the greatest deactivation of amygdala activity and had lower HF-HRV. This study suggests a complex influence of afferent autonomic signals on brain activity and emotion perception. In this line of thinking, the amygdala’s anticorrelation with HR can also be interpreted in the light of autonomic control. According toGianaros and Sheu (2009), the amygdala integrates different cortical processes (such as appraisal of fearful stimuli, cognition, memory, and attention) to adjust the cardiovascular reactivity and can be considered a part of the visceromotor system. The visceromotor system lacks a unified definition but can be viewed as mainly composed of the medial orbitofrontal cortex as described byPrice (1999)or, in broader terms, as defined, for example, inBarrett and Satpute (2013),Lane et al. (2013), orCritchley et al. (2011)to additionally contain the medial temporal gyrus, periaqueductal gray, subgenual anterior (sgACC), pregenual (pgACC) cingulate cortex, and amygdala. The primary role of this system is to regulate the autonomic nervous system by providing input to the hypothalamus (Gianaros & Sheu, 2009;Lane et al., 2013). In our main model, we found negative correlations with the medial orbitofrontal cortex, the sgACC and pgACC, as well as the medial temporal gyrus and bilateral amygdala (Fig. 6,Supplementary Fig. 5). Similarly, reversed correlations have been revealed by the HF-HRV model in the control analysis. Positive correlations of the sgACC and other regions of the visceromotor system with HF-HRV have also been reported byLane et al. (2013)and partially in the meta-analysis byBeissner et al. (2013). Therefore, our findings align with previous reports highlighting the role of these regions in autonomic control in response to stress and its associated affective response.

At the onset of each mental calculation block, we observed a strong increase in activity in the amygdala, as well as in the hippocampus and insula. For the amygdala and hippocampus, these peaks were stronger during the*Stress*phase compared with the two other phases. Initial strong increases in amygdala activity followed by a dampening of the neural response even during persistent stimulus presentation have been shown byVan Dillen et al. (2009), who observed amygdala activity at different cognitive loads after processing emotional stimuli. Amygdala activity peaked approximately 6 s after the onset of the emotional stimulus, and further activation might have been dampened by high cognitive load. This goes along with results from other studies (DeFraine, 2016;Mitchell et al., 2007) showing that cognitive load decreases the emotional response if emotions are not actively focused on due to task instructions.

In extension of van Dillen et al.’s findings, we observed significant initial increases in task-related activity at the block onset not only in the amygdala but also in the bilateral hippocampus and insula. The onset of each calculation block may be considered as an abrupt event that triggers arousal and has a putative negative valence. The amygdala and hippocampus, in particular, have been reported to be involved in emotional processing (Buhle et al., 2014;Davis & Whalen, 2001;Lindquist et al., 2012;Seymour & Dolan, 2008), while the anterior insula may play a more crucial role in integrating external stimuli and adjusting the large brain networks accordingly (Menon & Uddin, 2010;Molnar-Szakacs & Uddin, 2022). Thus, the initial peak at the block’s onset may reflect an immediate affective component of stress (Epel et al., 2018), that may then be reduced by high task demands.

Alternatively, this “initial peak” is an artifact due to physiological noise or movement. The beginnings of the calculation blocks may not only elevate activity in stress-related brain regions but could also lead to higher muscle tone and increased respiration, which may have been more pronounced during the*Stress*phase. We corrected for motion-related BOLD signal artifacts and motion-related drifts (two-steps: firstly, residualizing against motion correction parameters and their differentials, framewise displacement measures, linear detrending, and modeling images with intensity-based outliers features and, secondly, by adding the first five WM and CSF principal components to the first-level GLMs). Furthermore, a visual inspection of the regional timecourses showed that while there is also a peak in the CSF at the beginning of the block, it occurs after the peaks in the amygdala, hippocampus, and insula (Supplementary Fig. 6). Whether this initial peak represents an artifact, genuine neural activity, or a mixture of both, we confirmed that it did not drive our HR correlation results by including an additional peak-nuisance-regressor (10 s and 20 s). Essentially, this did not change our results (Supplementary Fig. 2). Moreover, our HR findings reflect a slower temporal pattern in the data (variations in the mean from block to block) than HR-associated artifacts within the blocks would subserve.

Finally, we found a positive correlation between HR and activity in the bilateral insula across all calculation blocks. Previous studies have reported increased insula activity in response to stress during comparable tasks (Berretz et al., 2021;Dedovic, D’Aguiar, & Pruessner, 2009;Wang et al., 2005;Zhong et al., 2019). The insula, particularly its anterior part, has been reported to be involved in autonomic regulation across a variety of tasks, including affective processing (Beissner et al., 2013). Moreover, the anterior insula has been found to be involved in emotion regulation (Zhang & Peng, 2023) and showed stronger activity during cognitive reappraisal in patients with mood disorders compared with healthy (Pico-Perez et al., 2017). The correlation of HR and insular activity under stress, which is evident in our results, may, therefore, reflect an affective component of the stress challenge.

Additionally, while the anterior part of the insula is the afferent hub of the salience network, balancing the activity of the executive control and default mode networks, and integrating internal and external stimuli (Menon & Uddin, 2010;Molnar-Szakacs & Uddin, 2022), the posterior insula is vital for interoception, the process of constant monitoring of internal signals from the periphery (Bonaz et al., 2021;Critchley, 2009;Pace-Schott et al., 2019). We observed a large cluster mainly in the bilateral anterior insula. Therefore, the positive correlation between anterior insula activity and HR could reflect the integration of perceived salient stimuli (calculations) and the monitoring of changes in HR occurring during the stress challenge. Following this idea, the lower correlation between left insula activity and HR during the*Stress*phase may be interpreted as a reduction in the integration of interoceptive processing (Supplementary Results&Supplementary Fig. 2).

One unresolved question is whether our results solely reflect the cognitive component of the calculations or the affective/emotional consequences of the psychosocial stressor (negative evaluative feedback). A closer inspection of varying HR across the task phases provides strong hints for combined (mental calculations and social evaluative feedback) stress-induced signal changes. After all, why do we observe similar maps in the*PreStress*and*PostStress*phase as in the*Stress*phase, if the maps specifically represent psychosocial stress? The highest HR during the*PreStress*phase was observed in the first mental calculation block (Supplementary Fig. 7), which seems plausible due to increased arousal caused by the beginning of the experiment as shown inZohdi et al. (2022). During the*Stress*phase, the highest HR occurred after the rest blocks with verbal feedback (one before the first*Stress*block, when participants were informed that their response would be ‘recorded’ from now on, and two negative social evaluative feedbacks during the*Stress*phase) (Supplementary Fig. 7), which may reflect a direct response to this kind of stressor. Finally, during the*PostStress*phase, the highest HR occurred in the first active block, most likely due to the downregulation that occurs after the*Stress*phase, which takes some time (Supplementary Fig. 7). Since cognitive load was constant within each task phase and HR (as an index of stress) varied, the results from our model can be interpreted in the light of affective processes (elicited by both the high demands of the mental calculations and the negative evaluative feedback) that go beyond cognitive load alone and highlight the significance of HR as a proxy of the stress response.

To identify clinically meaningful deviations in the stress response, an integration of baseline HR may be vital. Blunted cardiovascular stress reactivity is a hallmark of major depressive disorder (Schiweck et al., 2019) and several other psychological conditions and disorders (Beauchaine & Thayer, 2015;Quintana, Westlye, et al., 2016;Thayer et al., 2012). To this end, a meta-analytic study conducted byKemp et al. (2010)showed that HR variability decreases with increasing symptom severity in major depressive disorder, which raises the prospect of developing easy-accessible risk biomarkers to identify at-risk participants before diseases emerge.

## 5 Conclusion

Our results demonstrate the utility of integrating HR with fMRI to study the individual stress response during neuroimaging stress tasks. Through the modeling of individual HR traces, we observed regional BOLD fMRI coactivation in several brain regions typically associated with psychosocial stress. In conclusion, such combined models provide relevant complementary information to experimentally based task regressors and represent a straightforward method to compare various types of stress neuroimaging tasks, provided they include HR measures.

## Supplementary Material

Supplementary Material

## Data Availability

The datasets and code generated and/or analyzed during the current study contain clinical data and are not publicly available due to the protection of participants’ rights to privacy and data protection but are available from the corresponding author on reasonable request.
