# Supplementary material for: Capturing inter-individual variability in stress dynamics with heart rate traces reveals activity in the bilateral hippocampus, amygdala, and insula
Source: Imaging Neurosci (Camb). 2024 Dec 2;2:imag-2-00376. doi: 10.1162/imag_a_00376 (PMC12315724; doi:10.1162/imag_a_00376)
Supplement: Supplementary Material [file imag_a_00376-supp.pdf]

Supplementary Information

for the article entitled "Capturing inter-individual variability in stress dynamics with heart rate traces reveals activity in the bilateral hippocampus, amygdala and insula", Erhart et al.

Control Analysis:

The volumewise control analysis comprised a first-level GLM with individual mean HR values per volume instead of a blockwise HR arranged in one regressor. As for the main model, a one-sample t-test was performed on the parametric modulator at the group level.

To examine the effect of the experimental phase, an additional GLM captured the five active blocks within each task phase (PreStress, Stress and PostStress) as separate regressors with individual blockwise mean HR as a parametric modulator (phasewise model with mean HR). The Random effect second-level analysis forwarded the differential first level t-contrast between Stress and aggregated PreStress and PostStress (contrast vector [-1 +2 -1]) to the group level.

Furthermore, we used HF-HRV as a parametric modulation instead of HR and integrated it similarly to our main model (one regressor with 15 task-blocks and average HF-HRV per block as the parametric modulation). Although HR and metrics of heart rate variability such as HF-HRV are anticorrelated, (Kazmin et al, 2016; in our data: $r=-.51$, $p<0.001$), we additionally included it as a parametric modulation since HF-HRV has previously been associated with stress, panic, and anxiety (Chuang, 2009; Hughes, 2000; Kazmi et al., 2016; Mulcahy et al., 2019; Shaffer & Ginsberg, 2017).

For the HF-HRV model, the random effects second-level analysis was a one-sample-t-test of the contrast of the parametric modulator forwarded to the group level.

Supplementary results:

The differential contrast for the phasewise model with mean HR comparing the positive correlation with the parametric modulator of the combined *PreStress/PostStress* phase with the *Stress* phase revealed a significantly stronger correlation in the bilateral middle temporal gyri, the right parahippocampal area, and the bilateral middle occipital lobe and bilateral lingual gyri during the *Stress* phase and a weaker correlation in the left insula, the bilateral superior temporal gyri and bilateral supplementary motor area during the *Stress* phase (**Supplementary Figure 2, Supplementary Table 1**). The results from the HF-HRV model showed a map that was similar to our main model, as expected, but in the opposite direction because HR and HF-HRV were anti-correlated (e.g. the amygdala and hippocampus now showed a positive correlation) compared with the main model (**Supplementary Figure 2, Supplementary Table 1**). Positive correlations were observed with the bilateral orbitofrontal cortex, caudate, putamen, bilateral amygdala, and hippocampus as well as with the temporal pole and right pre- and postcentral gyri and right pregenual and subgenual anterior cingulate cortex. Negative correlations were found in the bilateral frontal inferior gyri and left cerebellum.

Supplementary Table 1: Clustertable for control analysis. Model name, coordinates of the peak voxel, cluster size (k), region labels as revealed by the AAL3 toolbox including % voxel (% of k) of the cluster labeled with the respective name, p-value and t-values of the peak voxel. Note: only region labels > 10% of the cluster are presented.

Model and contrast	Peak coordinates			Label	k	% of k	p	t
	x	y	z					
Volumewise HR Model: +1	24	-62	46	Right inferior parietal lobe	1761	24.19	0.000	10.82
				Right superior lobe		22.15		
				Right superior occipital lobe		12.66		
	-26	-70	36	Left inferior parietal lobe	1909	33.32	0.000	10.32

				Left superior parietal lobe		30.70		
				Left middle occipital lobe		16.66		
	-44	2	30	Left precentral gyrus	288	84.38	0.000	9.27
				Left inferior frontal gyrus, opercular part		14.93		
	-50	-66	-12	Left inferior occipital lobe	757	43.86	0.000	8.55
				Left inferior temporal lobe		20.08		
	28	-94	-8	Right inferior occipital lobe	354	72.32	0.000	7.92
				Right lingual gyrus		10.73		
	48	6	28	Right precentral gyrus	74	67.57	0.000	7.54
				Frontal_Inf_Oper_R		32.43		
	42	-60	-12	Right superior frontal gyrus	36	22.22	0.000	7.27
	56	-48	-16	Right inferior temporal gyrus	30	48.75	0.000	6.93
				Right fusiform area		20.00		
	-28	-10	48	Left precentral gyrus	29	48.28	0.000	6.71
				Left superior frontal gyrus		41.38		
				Left middle frontal gyrus		10.34		
Volumewise HR Model: -1	-8	-94	20	Left cuneus	3369	28.91	0.000	11.06
				Right cuneus		15.08		
				Right lingual gyrus		14.49		
	54	-4	-18	Right middle temporal lobe	109	69.72	0.000	8.16
				Right superior temporal lobe		29.36		
	2	50	-14	left medial frontal gyrus, medial orbital	696	31.32	0.000	8.16
				right medial frontal gyrus, medial orbital		18.97		
				Left rectus		15.95		
				Right rectus		15.09		
	-60	-38	0	Left middle temporal lobe	310	99.35	0.000	8.05
	-2	-48	34	Left precuneus	110	40.00	0.000	7.82
				Left posterior cingulate cortex		35.45		
				Left middle cingulate cortex		21.82		
	64	-24	12	Right superior temporal lobe	381	47.24	0.000	7.8
				Right rolandic operculum		29.92		

				Right Heschel's gyrus		12.60		
	-62	-30	12	Left superior temporal lobe	48	100	0.000	7.46
	62	-32	0	Right middle temporal lone	80	82.50	0.000	7.32
				Right superior temporal lobe		17.50		
	-44	-76	38	Left angular gyrus	132	62.12	0.000	7.05
	38	-20	4	Right insula		73.68	0.000	6.93
Three-phase Model with mean HR: -1 +2 -1	48	-64	2	Right middle temporal lobe	988	45.55	0.000	12.88
				Right middle occipotal lobe		27.33		
				Right inferior temporal lobe		12.35		
	-42	-74	4	Left middle occipital lobe	483	71.22	0.000	12.18
				Left middle temporal lobe		13.66		
	-6	-90	-10	Right fusiform area	1872	32.26	0.000	10.99
				Right lingual gyrus		22.81		
				Left calcarine sulcus		14.10		
				Left fusiform area		11.75		
				Left lingual gyrus		11.75		
	8	-32	44	Middle cingulate cortex	46	95.65	0.000	6.93
Three-phase Model with mean HR: +1 -2 +1	-56	-24	2	Left middle temporale lobe	2878	50.87	0.000	13.93
				Left superior temporal lobe		49.13		
	62	-22	4	Right superior temporal lobe	2369	93.20	0.000	11.79
	0	14	58	Left Supplementary motor area	117	59.81	0.000	6.54
				Right supplementary motor area		39.42		
	-42	22	-4	Left inferior frontal gyrus, orbital part	43	45.05	0.000	6.14
				Left inferior frontal gyrus, pars triangularis		27.77		
				Left insula		24.66		
Task-regressor based Model: -1 +2 -1	-6	-90	-8	Left middle occipital lobe	10789	13.98	0.000	15.58
				Right fusiform area		10.7		
	6	52	54	Right precuneus	33	100	0.001	5.72
	4	-64	-20	vermis	32	100	0.004	5.51
	-60	-22	-2	Middle temporal gyrus	576	75.73	0.000	7.54

Task-regressor based Model: +1 -2 +1	44	-22	10	Right superior temporal gyrus	71	31.46	0.005	5.3
				Right Heschel's gyrus		29.51		
				Right rolandic operculum		19.22		
				Right insula		14.95		
HF-HRV Model: +1	44	-64	2	Right middle temporal gyrus	451	75.17	0.000	9.99
				Right middle occipital gyrus		11.71		
	-42	-74	6	Left middle occipital gyrus	201	86.07	0.000	8.94
	-4	68	-14	Left orbitofrontal cortex	528	22.73	0.000	8.93
				Right orbitofrontal cortex		22.54		
				Left middle temporal gyrus		13.96		
	52	-24	46	Right postcentral gyrus	837	56.63	0.000	8.75
				Right precentral gyrus		29.99		
	-8	-32	-4	Left lingual gyrus	71	29.58	0.000	8.19
HF-HRV Model: -1	58	34	-14	Right inferior frontal gyrus	465	12.7	0.000	8.98
	-66	12	14	Left inferior frontal operculum	198	11.5	0.000	6.95
	-32	-68	-42	Cerebelum_Crus2_L	41	75.61	0.000	6.92
				Cerebelum_Crus2_R		24.39		

Supplementary Table 2: Clustertable for control analysis. Model name, coordinates of the peak voxel, clustersize (k), region labels as revealed by the AAL3 toolbox including % voxel (% of k) of the cluster labeled with the respective name, p-value and t-values of the peak voxel. Note: only region labels > 10% of the cluster are presented.

Model 10s: +1	-6	6	52	Left precentral gyrus	3819	33.49	0.000	14.08
				Left supplementary motor area		12.54		
	-30	18	8	Left insula	375	86.93	0.000	12.22
	28	42	-26	Right anterior orbital gyrus	205	46.34	0.000	11.45
	46	6	28	Right precentral gyrus	1650	25.21	0.000	11.24
				Right inferior frontal operculum		24.55		
				Right superior frontal gyrus		22.97		
	8	70	-10	Right superior frontal gyrus	893	46.25	0.000	11.06
				Left anterior orbital gyrus		11.76		
				Right orbito-medial frontal gyrus		10.75		

	42	42	30	Right middle frontal gyrus	782	73.79	0.000	10.67
				Right inferior frontal gyrus, pars triangularis		21.61		
	32	24	4	Right insula	341	71.85	0.000	10.51
	-12	-60	-50	Left cerebrum	28	53.57	0.000	7.2
	40	-54	-54	Right cerebrum	25	100	0.004	6.12
	50	-68	34	Right angular gyrus	1872	50.00	0.000	14.36
				Right middle temporal gyrus		22.01		
	24	-86	34	Cerebelum_Crus2_R	927	52.64	0.000	12.18
				Cerebelum_Crus1_R		33.98		
	-20	-88	-36	Cerebelum_Crus2_L	514	53.11	0.000	10.98
				Left crus I of cerebellar hemisphere		34.05		
	8	-52	-44	Right cerebrum	166	77.11	0.002	10.91
				Left cerebrum		15.66		
	14	-74	-2	Right lingual gyrus	41	100	0.002	7.21
Model 20s: +1	-44	-62	-10	Left inferior parietal gyrus	8779	12.76	0.000	12.09
				Left middle occipital gyrus		11.52		
	28	42	-26	Right anterior orbital gyrus	120	30.83	0.000	10.6
	-44	0	26	Left precentral gyrus	1082	65.25	0.000	10.53
				Left inferior frontal operculum		11.65		
	8	70	-10	Right orbito-medial frontal gyrus	602	16.28	0.000	10.08
				left orbito-medial frontal gyrus		13.62		
				Right superior frontal gyrus		13.12		
	54	-48	-12	Right fusiform area	1749	22.87	0.000	9.83
				Right inferior temporal gyrus		22.06		
				Right inferior occipital gyrus		13.63		
				Right cerebrum		10.25		
	-8	8	52	Right supplementary motor area	1863	41.20	0.000	9.08
				Left supplementary motor area		40.53		
				Right middle cingulate cortex		12.92		
	-32	34	-32	left anterior orbital gyrus	449	16.76	0.000	8.83

	-30	16	8	Left insula	173	98.48	0.000	8.79
	46	6	28	Right frontal inferior operculum	132	38.32	0.001	8.76
				Right precentral gyrus		36.53		
				Right suprior frontal gyrus		11.78		
	46	42	28	Right middle frontal gyrus	501	86.02	0.000	8.37
-1	-42	-68	28	Left angular gyrus	1345	42.30	0.000	10.15
				Left middle temporal gyrus		18.22		
				Left middle occipital gyrus		14.57		
	50	-66	34	Right angular gyrus	995	60.60	0.000	9.72
				Right middle temporal gyrus		24.02		
	34	-84	-36	Right crus II of cerebellar hemisphere	765	44.44	0.000	9.16
				Right crus I of cerebellar hemisphere		39.48		
	-24	-88	-36	left crus II of cerebellar hemisphere	415	51.81	0.000	9.14
				Left crus I of cerebellar hemisphere		18.55		
	8	-52	-44	Right cerebrum	38	92.11	0.000	6.98
	44	-32	20	Right rolandic operculum	85	50.59	0.000	6.77
				Right superior temporal gyrus		25.88		
				Right supramarginal gyrus		20.00		
	-36	12	56	Left middle temporal gyrus	40	100	0.003	6.65
	36	-14	18	Right insula	29	68.97	0.001	6.28
				Right rolandic operculum		27.59		

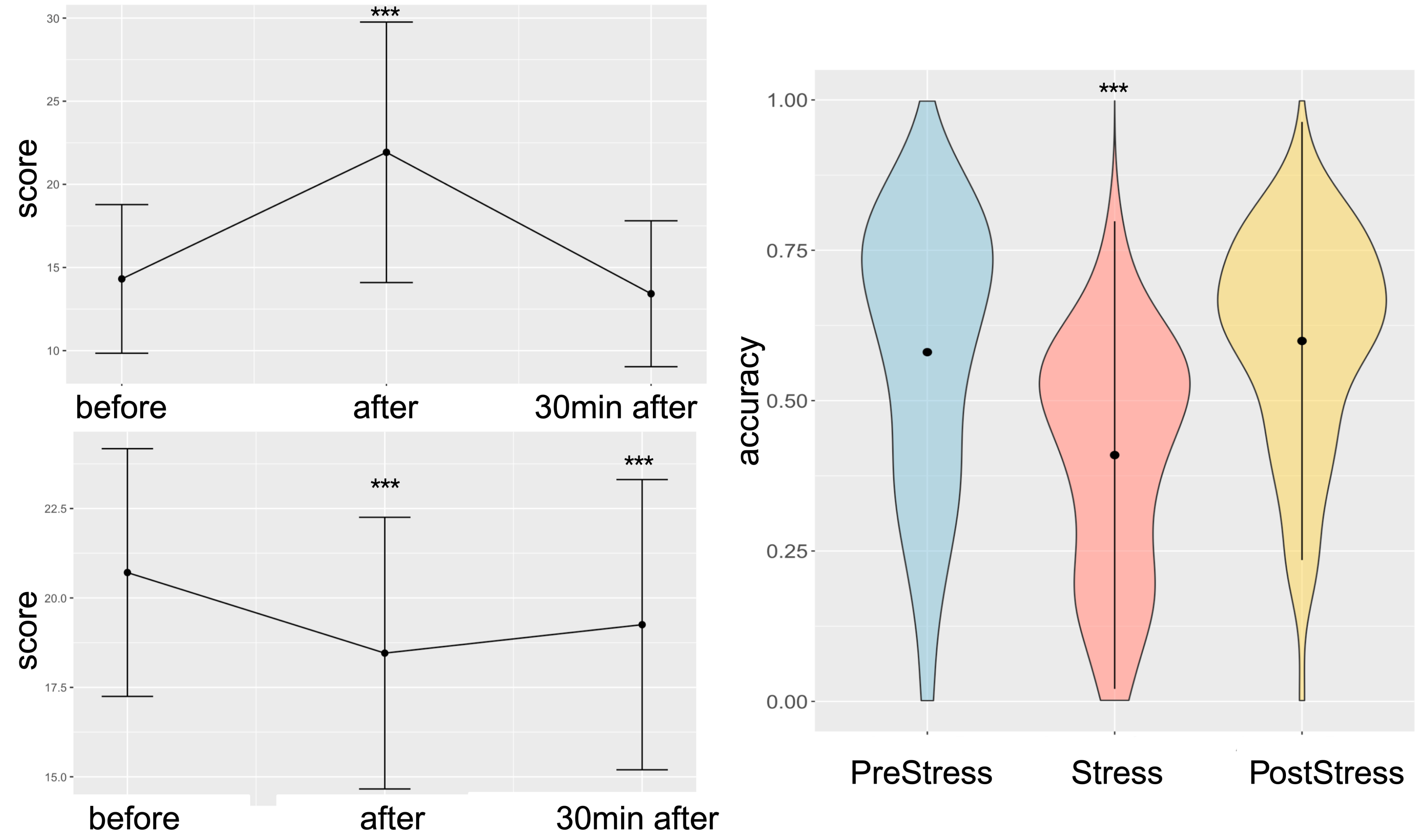

Supplementary Figure 1. Results of subjective feelings and behavioral performance during and after the Imaging Stress Task. Left: upper panel shows mean positive and lower panel mean negative emotions (measured with BSKE before, after and 30min after the task) with standard deviations as errorbars. Negative emotions were significantly higher after the task and recoverd after 30min, while positive emotions significantly decreased and did not recover 30min after the task. Right: accuracy during the Imaging Stress Task; black dots represent the mean and black lines the 25% and 75% of the distribution. Accuracy was significantly lower during the stress compared to the two other phases.

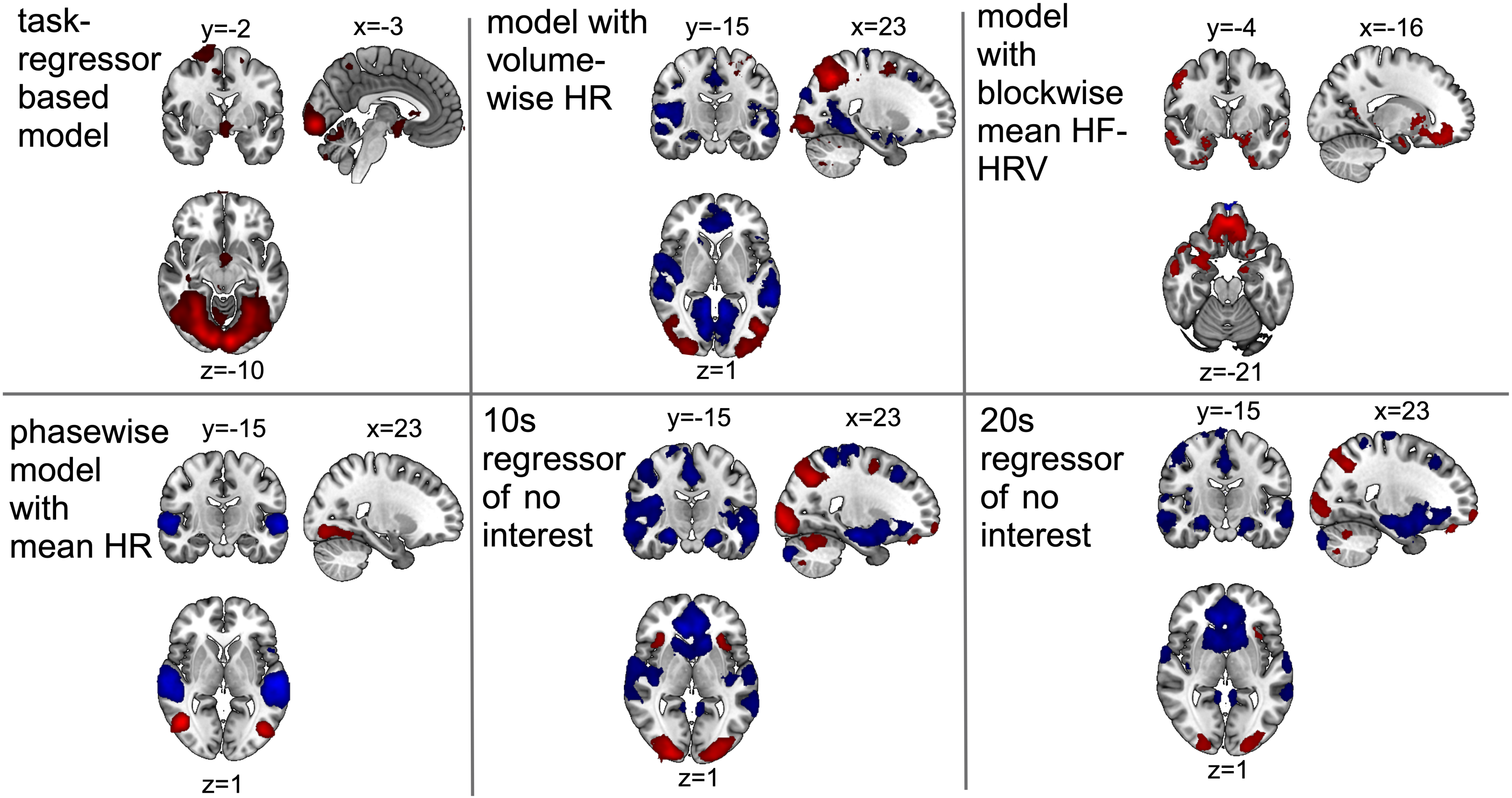

Supplementary figure 2. Results of all control analysis. Control analysis for the different options of integrating heart rates or high-frequency power (HF-HRV) and the task-regressor based model, and the two models controling for the BOLD peak at onset of each active block in bottom row (last two). Top (from left to right): Depicted are the block-amplitude contrast between *Pre-* and *PostStress* phases against the *Stress* phase; neural correlates with a volumewise parametric modulator across all 15 blocks; neural correlates with a blockwise HF-HRV parametric modulator across all 15 blocks; Bottom: 3 task phases in separated regressors with mean blockwise HR as parametric modulator; model with a 10s regressor of no interest and with 20s regressor of no interest at each onset of the blocks. Decreased default mode network activity and negative correlations between HR and activity in bilateral hippocampus and amygdala remain. All maps are thresholded at $p_{voxel.FWE}<0.05$ and cluster extent>25. red= *Stress* > *PreStress*/ *PostStress* or positive correlation with HR/HF-HRV. blue= reversed or negative correlation.

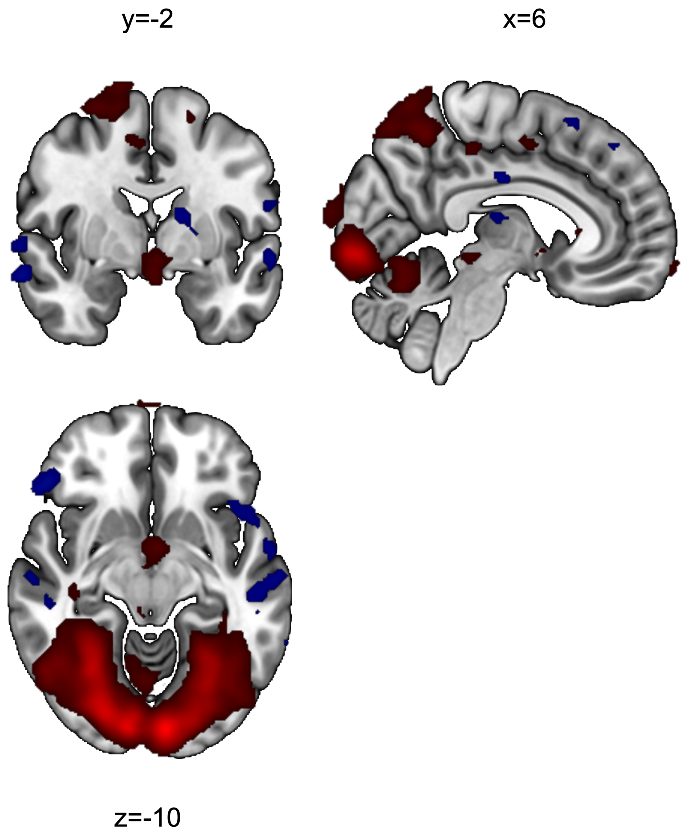

Supplementary Figure 3. Classical contrast of Model (A) at lower threshold. p<0.001 (uncorrected), k>25 voxel.

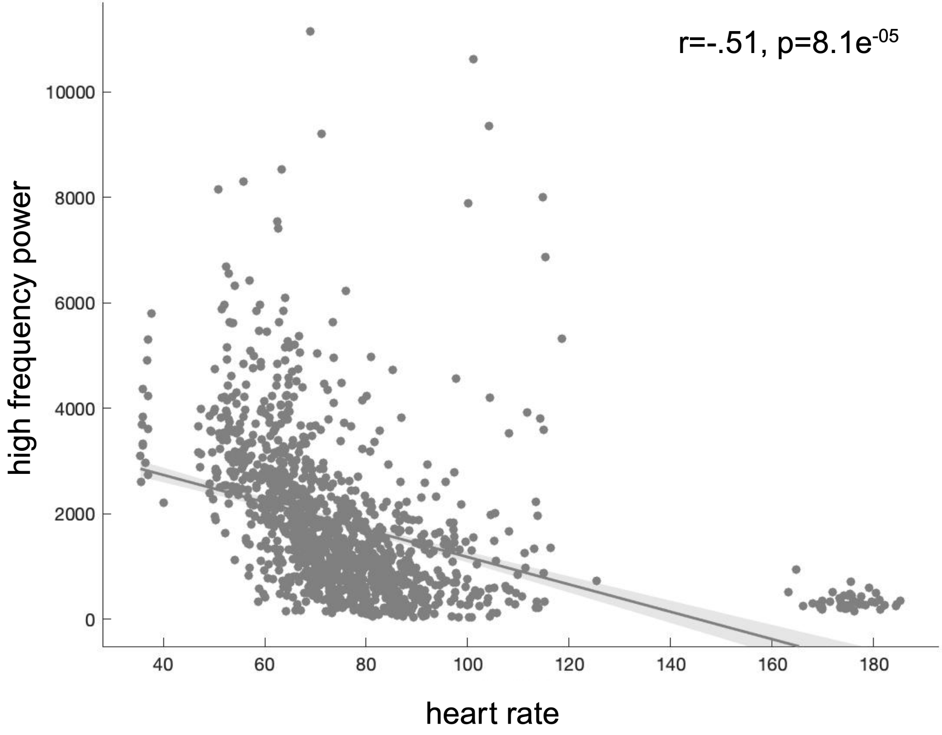

Supplementary figure 4: Negative correlatiion of blockwise heart rate and high-frequency power (0.15-0.4 Hz) in our sample.

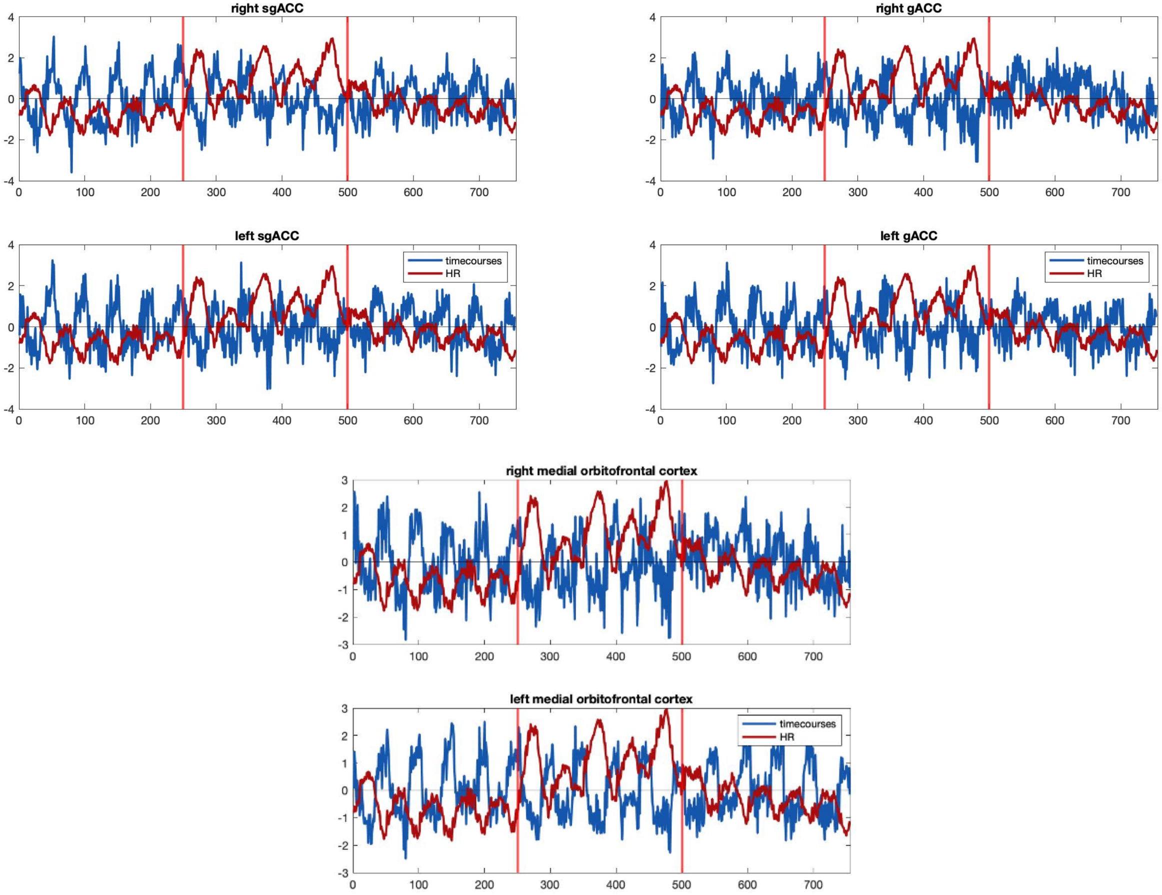

Supplementary Figure 5. Mean z-scored timecourses (first principal component) for the right (top) and left (bottom) sgACC, gACC and medial orbitofrontal cortex peak voxels with a 5mm sphere around it (blue) together with the mean heart rate (red).

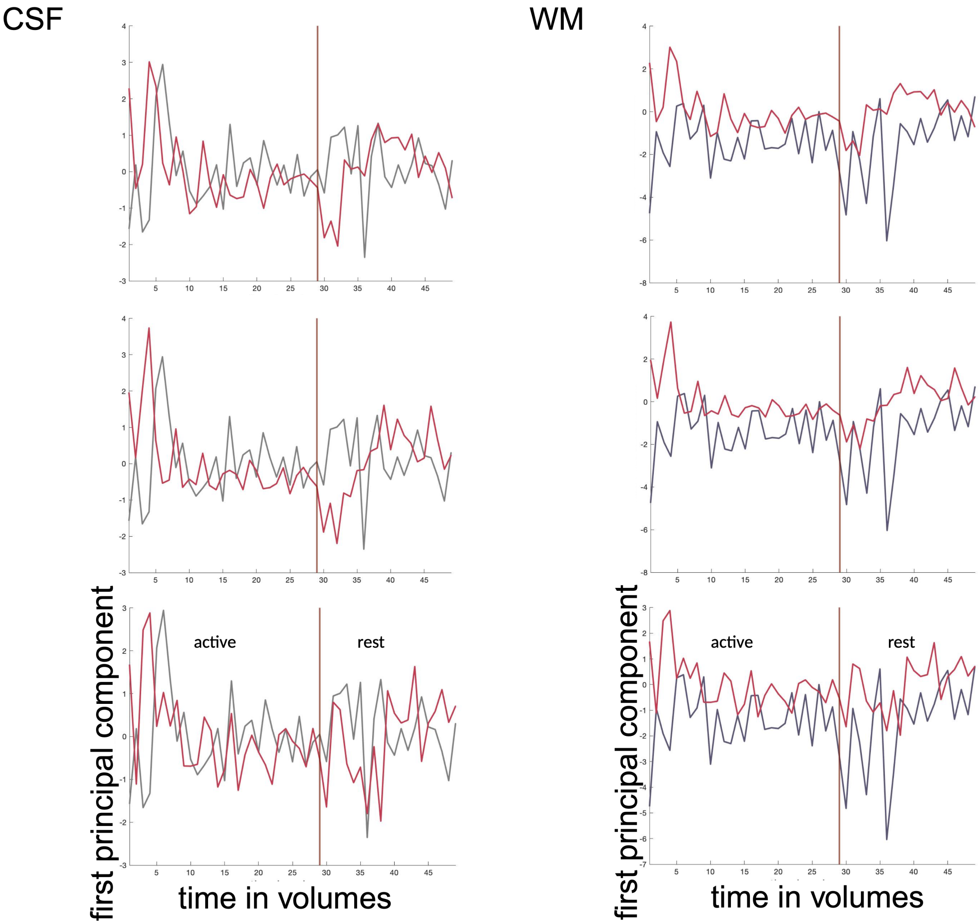

Supplementary Figure 6. Mean z-transformed regional timecourses and nuisance regressors (first principal components). Grey lines represent the z-transformed mean first principal component for the cerebrospinal fluid (left) and white matter (right) and red lines mean regional time courses across all blocks and phases (left hippocampus, left amygdala, left insula; from top to bottom). Peaks in activity preceded CSF peaks and were not related to WM changes. Similar results were obtained for the right hemisphere of all three ROIs (not shown).

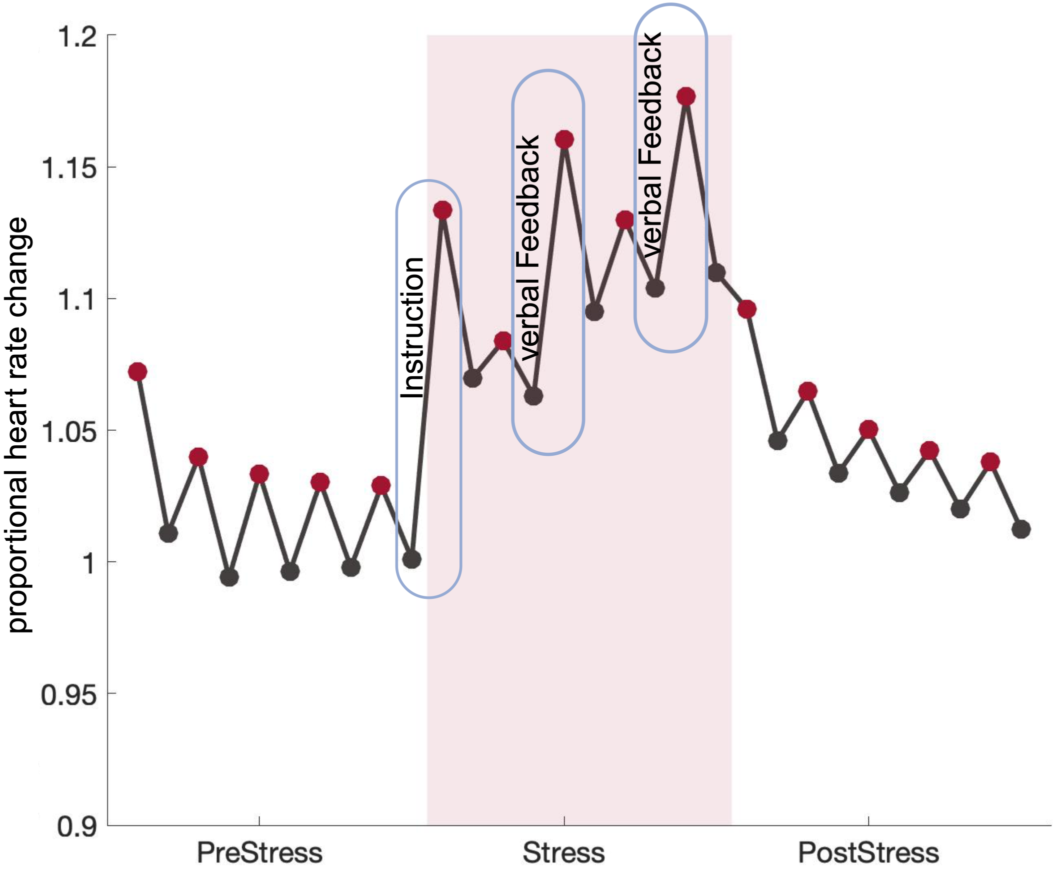

Supplementary Figure 7. Heart rate changes and task stressors. Heart rate increases were mainly observed after the rest blocks during which instructions or verbal feedback were given. Red dots represent the mean heart rates per active block normalized to the mean rest block PreStress heart rate; black dots represent the same for rest blocks; pink shaded area marks the stress phase; ellipses represent the rest blocks during which instructions or negative verbal feedback were provided and the following heart rate changes in the active phase.
